# Synthesis, Supramolecular Assembly, and Hydrogelation of Poly(amino ester) ABA Triblock Copolymers

**DOI:** 10.1021/acs.biomac.5c01828

**Published:** 2025-12-15

**Authors:** Chloé Pascouau, Kamila Wittek, Jessica Erlenbusch, Sebastian Becker, Jochen Fischer-Schuch, Pablo G. Argudo, Pol Besenius

**Affiliations:** † Department of Chemistry, 9182 Johannes Gutenberg-University Mainz, Duesbergweg 10-14, D-55128 Mainz, Germany; ‡ Institut für Biotechnologie und Wirkstoff-Forschung gGmbH, Hanns-Dieter-Hüsch-Weg 17, 55128 Mainz, Germany; § Department of Molecular Spectroscopy, 28308 Max Planck Institute for Polymer Research, Ackermannweg 10, 55128 Mainz, Germany

## Abstract

Poly­(amino esters) derived from *N*-acylated-1,4-oxazepan-7-ones (OxPs) emerge as promising candidates in the development of new and degradable amphiphiles for hydrogel preparation and delivery formulations. Here, the synthesis of amphiphilic triblock copolymers by ring-opening copolymerization of OxP monomers with various pendant chains is reported. Copolymerization using organocatalysts and a bifunctional initiator afforded neutral P­(OxP_Me_)-*b*-P­(OxP_Bn_)-*b*-P­(OxP_Me_) and cationic P­(OxP_NH2_^+^)-*b*-P­(OxP_Bn_)-*b*-P­(OxP_NH2_^+^) amphiphilic triblock copolymers with controlled molar masses ranging from 4,600 to 8,500 g/mol and narrow dispersities (Đ ≤ 1.21). A panel of polymers with various block lengths and compositions was synthesized. Their self-assembly in water revealed the formation of nanostructures, including worm-like or spherical morphologies. Modulation of the copolymer composition and concentration enables control over hydrogelation and its macroscopic properties. Finally, we investigated the formulation of a hydrophobic fungicide and its inhibitory effect on spore proliferation, which shows great promise as dispensable and biodegradable hydrogel formulation for agrochemical applications.

## Graphical abstract

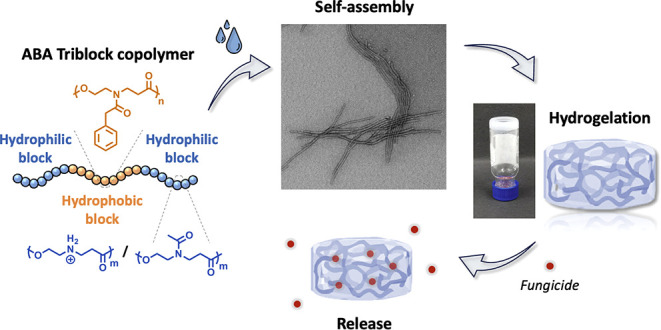

## Introduction

Polymeric delivery systems have attracted significant research interest over the last years for the development of new materials in the biomedical and agrochemical fields.^1–6^ Progress in controlled polymerization, along with the availability of a variety of precursors, has enabled the design of well-defined block copolymers with tailored topologies, compositions, and tunable properties.^7–10^ Thanks to these advances, amphiphilic block copolymers have been extensively studied as vehicles and carriers for drug delivery owing to their numerous properties, including high loading capacity, biocompatibility, controlled release, and improved stability.^11–13^ These materials have demonstrated a remarkable ability to self-assemble into diverse nanostructures, such as micelles, spherical or rod-like nanostructures, as well as hydrogels.^13–16^ In particular, the development of polymeric hydrogels is of great interest due to their high water retention capacity and good biocompatibility.^17–20^ Unlike their covalently cross-linked analogues, the driving force of supramolecular hydrogel formation lies in reversible noncovalent interactions, including hydrogen bonding, Coulomb interactions, and hydrophobic effects, which result in a more dynamic and adaptable network. Numerous studies have demonstrated the efficient use of amphiphilic triblock copolymers in the formation of supramolecular hydrogels; however, these materials often lack degradability.^21–26^ Therefore, the development of materials that combine biodegradability and biocompatibility is of significant interest.

Poly­(amino ester)­s (PAEs) have shown particular relevance in the development of degradable polymers in recent years.^27–30^ The interesting feature of PAEs lies in the combination of polymer backbone ester linkages for degradability and amine or amide derivatives, which provide side-chain functionalities and pH sensitivity. Thanks to their biodegradable and biocompatible properties, PAEs appeared as promising candidates for biomedical applications, including gene and drug delivery and bioimaging.^31–38^ In addition, the possibility to yield polycationic PAEs with a charged block enables electrostatic interactions with oppositely charged compounds, such as DNA and RNA, which is of great interest in the design of polyplexes.^39–41^ Therefore, PAEs facilitate the complexation/release and delivery of various pharmacologically active compounds. By introducing diverse side chains along the polymer backbone, specific applications can also be targeted through postmodification reactions.^42–46^

For decades, the synthesis of poly­(β-amino ester)­s was reported through step-growth polymerization protocols such as Michael addition and polycondensation reactions, which allow the use of numerous vinyl monomer precursors and primary amines.^30,39,47–49^ The use of chain-growth polymerization methods for the controlled synthesis of poly­(β-amino ester)­s has only recently been reported.^50–54^ Specifically, the organocatalytic ring-opening polymerization (ROP) of *N*-acylated-1,4-oxazepan-7-ones (OxPs) at room temperature gave access to a metal-free synthesis of degradable PAEs with controlled molar masses and narrow dispersities.^55,56^ The possibility to afford polymers with diverse chemical properties through the incorporation of functional monomers enables the modulation of structure–function relationships. By adjustment of the OxP monomer and its pendant side-chain, polymers with different properties, such as water solubility, were achieved. As an example, the homopolymer obtained by ROP of OxP bearing a 2-phenylacetyl group (OxP_Bn_) is hydrophobic, while the OxP monomer with an acetyl side chain (OxP_Me_) affords a water-soluble homopolymer.^55^ In addition, the ROP of Boc-protected OxP monomer (OxP_Boc_) and subsequent deprotection enabled the synthesis of hydrophilic and polycationic PAEs.^50^ Amphiphilic block copolymers can potentially be designed by combining hydrophilic and hydrophobic blocks for self-assembly into nanostructures of controlled shapes and size. The variation of the copolymer composition would provide opportunities to modulate the packing parameters and supramolecular morphologies.^57,58^ These strategies have not been used yet due to the lack of synthetic accessibility, given that previous syntheses have largely used polycondensation procedures.

In this work, a wide range of ABA triblock copolymers based on PAEs was synthesized by ROP of OxP monomers with acetyl (OxP_Me_), Boc (OxP_Boc_), and 2-phenylacetyl (OxP_Bn_) pendant groups. P­(OxP_Me_)-*b*-P­(OxP_Bn_)-*b*-P­(OxP_Me_) and polycationic P­(OxP_NH2_^+^)-*b*-P­(OxP_Bn_)-*b*-P­(OxP_NH2_^+^) amphiphilic triblock copolymers were obtained using 1,4-benzenedimethanol (DiOH) as a bifunctional initiator and 1,8-diazabicyclo[5.4.0]­undec-7-ene (DBU) and 1-(3,5-bis­(trifluoromethyl)­phenyl)-3-cyclohexyl thiourea (TU) as organocatalysts. The combination of DBU and TU previously demonstrated high monomer conversion rates (≥89%) in short reaction times (80–100 min), and polymers with narrow dispersites (Đ ≤ 1.13).^56^ The self-assembly of the block copolymers into nanostructures of different shapes and sizes was investigated in water and correlated to their macromolecular composition, block size, and hydrophilic/hydrophobic balance. Hydrogelation in concentrated aqueous media enabled investigations into the mechanical properties of the hydrogels as a function of the nature of the block copolymer and the organic weight content. Finally, the potential of using PAEs-based hydrogels as an application platform was evaluated by formulating a water-insoluble fungicide into the hydrogels and studying its inhibitory effect on spore proliferation of the fungus *Phaeomoniella chlamydosporum*.

## Experimental Section

### Materials

All dry solvents used for polymerizations were purchased from *Thermo Fisher Scientific* with a purity of 99.8%. All chemicals used for monomer synthesis and polymerizations were obtained from *TCI Chemicals, Sigma-Aldrich, Apollo Scientific, Avantor ScienceCentral*, and *abcr*, with a purity of at least 98% with the exception of *m*-chlor perbenzoic acid (70%). The monomers and DiOH were dried via azeotropic distillation using dry toluene several times and were kept under dynamic vacuum overnight. DBU was dried over CaH_2_ and stirred overnight, followed by a vacuum-distillation. All dry compounds were stored in an argon-filled glovebox. Water used for sample preparation was deionized and purified using a PURELAB flex 4 purification system by *Veolia Water Solutions* *& Technologies*.

### Monomer Synthesis

4-(2-Phenylacetyl)-1,4-oxazepan-7-one (OxP_Bn_),^55^ 4-acetyl-1,4-oxazepan-7-one (OxP_Me_),^56^ and 4-*tert*-butoxycarbonyl-1,4-oxazepan-7-one (OxP_Boc_)^56^ monomers were synthesized according to previously reported methods.

Briefly, OxP_Boc_ was synthesized by Bayer-Villiger ring-expansion of 1-*tert*-butoxycarbonyl-4-piperidone (PBoc). m-CPBA (70%; 1.5 equiv; 73.4 mmol; 12.7 g) was solubilized in DCM (80 mL) and added dropwise to a cold solution of PBoc (1 equiv; 37.6 mmol; 7.5 g) in DCM (30 mL). The mixture was stirred overnight at room temperature under an argon atmosphere. The precipitates formed during the reaction were removed by filtration, and 0.5 equiv of m-CPBA was added. The mixture was stirred overnight under an argon atmosphere at room temperature. After complete consumption of the reagent, the organic phase was washed first with Na_2_S_2_O_3_/NaHCO_3_ solutions (50/50), then with a NaHCO_3_ solution until the aqueous phase was no longer colored, and finally with distilled water. The organic phases were dried over MgSO_4_, filtered, and the solvent was evaporated under reduced pressure to afford the product as a slightly yellow solid. OxP_Boc_ was purified by silica gel chromatography (cyclohexane/ethyl acetate = 1:1). Yield: 65%, colorless solid. ^1^H NMR (CDCl_3_, 294 K) δ/ppm = 4.25 (m, 2H, COOCH_2_), 3.77 (m, 2H, COOCH_2_CH_2_N), 3.66 (m, 2H, NCH_2_CH_2_COO), 2.80 (m, 2H, CH_2_COO), 1.47 (s, 9H, CH_3_) (Figure S1).

OxP_Me_ was obtained by a two-step synthesis starting from OxP_Boc_. First, Boc deprotection was performed for the synthesis of 1,4-oxazepan-7-one trifluoroacetate salt (OxP_TFA_). A TFA/DCM solution (5/2 mL) was added to a solution of OxP_Boc_ (5 g) in DCM (4 mL), and the mixture was stirred at room temperature for 45 min. TFA and solvent were evaporated, and the yellow viscous solid was dissolved in a small amount of DCM and precipitated twice in diethyl ether. The solid compound was isolated and dried under reduced pressure to yield OxP_TFA_ as a white solid.

OxP_TFA_ was then acetylated to afford OxP_Me_. K_2_CO_3_ (3 eq; 29.7 mmol; 4.11 g) and OxP_TFA_ (1 eq; 9.91 mmol; 2.72 g) were first mixed in DCM (50 mL), affording a heterogeneous solution. After 10 min, acetyl chloride (2 equiv; 19.8 mmol; 1.42 mL) was added to the solution, and the mixture was stirred for 16 h at room temperature under an argon atmosphere. The mixture was then filtered, and the solvent was evaporated under reduced pressure, yielding the product as a white solid. OxP_Me_ was purified by silica gel chromatography (DCM/methanol = 50:1). Yield: 70%, colorless solid. ^1^H NMR (CDCl_3_, 294 K) δ/ppm = 4.30–4.26 (m, 2H, COOCH_2_), 3.95–3.67 (m, 4H, N­(CH_2_)_2_), 2.85–2.81 (m, 2H, CH_2_COO), 2.16 (d, 3H, CH_3_) (Figure S2).

OxP_Bn_ was synthesized by a two-step synthesis. 4-Piperidone hydrochloride monohydrate (1.0 eq., 0.013 mol, 2.0 g) and K_2_CO_3_ (3.0 eq., 0.039 mol, 5.4 g) were suspended in DCM (66 mL) and vigorously stirred for 5 min at room temperature in a nitrogen atmosphere. Phenylacetyl chloride (1.5 equiv, 0.020 mol, 3.0 g) was then added, and the reaction was stirred for 20 h. The reaction was quenched by adding a 1 M NaOH solution (30 mL) under cooling using an ice bath, and stirred for a further 30 min. The reaction mixture was transferred into an extraction funnel, and the aqueous phase was extracted with DCM (4 × 10 mL). The combined organic phases were dried over Na_2_SO_4_, filtered, and the solvent was evaporated under reduced pressure, yielding the desired product as a yellow oil. Afterward, Baeyer–Villiger oxidation was performed. *m*-CPBA (2.0 equiv, 0.026 mol, 4.5 g) was dissolved in DCM (50 mL) while stirring and cooled down to 0 °C. 1-Phenacetyl-4-piperidone (1.0 eq., 0.013 mol, 2.8 g) was dissolved in DCM (15 mL) and added dropwise to the cooled reaction mixture. The solution was warmed to room temperature and stirred for 14 h. *m*-CPBA (0.5 equiv, 0.006 mol, 1.1 g) was added as a solid, and the reaction was stirred for a further 24 h. Half-saturated thiosulfate solution (10 mL) was added to the reaction mixture to neutralize residual peroxides, and the mixture was stirred for 30 min. The solution was then transferred into an extraction funnel, and the organic phase was washed with saturated NaHCO_3_-solution (5 × 20 mL) and saturated NaCl-solution (3 × 20 mL). The organic phase was dried over Na_2_SO_4_, filtered, and evaporated under reduced pressure. Column chromatography was performed for further purification (SiO_2_, 1:1 cyclohexane/ethyl acetate, 1:1). Yield: 73%, colorless viscous liquid. ^1^H NMR (CDCl_3_, 294 K) δ/ppm = 7.35–7.25 (m, 5H, phenyl), 4.19–3.63 (m, 8H, COOCH_2_, N­(CH_2_)_2_, CH_2_Ph), 2.73–2.38 (2m, 2H, CH_2_COO) (Figure S3).

### General Polymerization Method

All polymerizations were performed inside an argon-filled glovebox. Here is an example of the polymerization of OxP_Bn_ (M1) and OxP_Me_ (M2) using DBU (C1) and TU (C2) as catalysts and DiOH (I) as initiator with the following parameters: M1/M2/C1/C2/I ratio = 8/18/6/6/1; DCM (*V*_M1_ = 0.235 mL and *V*_M2_ = 0.680 mL); [M1] = 1 M and [M2] = 1 M; room temperature (RT). In a first vial, DBU (6 eq; 0.257 mmol; 38.4 μL), TU (6 eq; 0.257 mmol; 95.3 mg), DiOH (1 eq; 0.0429 mmol; 5.9 mg), and one part of the solvent (DCM *V*_M1_) were mixed and stirred for 10 min. Thirty μL of THF was added for better solubilization of the initiator. Then, the first monomer was solubilized with the other part of the solvent (DCM *V*_M1_) in a separate vial. After monomer solubilization, the initiator/catalyst solution was added to the vial containing the monomer. The mixture was stirred for 90 min, and an aliquot of the reaction was taken. After complete conversion of the first monomer, the second monomer was solubilized (DCM *V*_M2_) and added to the reaction. After 90 min, the vial was removed from the glovebox, and the reaction was quenched with acetic acid. Another aliquot was taken, and the solution was precipitated twice in a mixture of ethanol and diethyl ether (15/85). The OxP_Boc_ and OxP_Bn_-based copolymers were precipitated in diethyl ether. The reaction times for the polymerization of the first and second blocks were adapted as a function of polymer chain length, ranging from 60 to 180 min. After complete polymerization of OxP_Boc_ and OxP_Bn_-based polymers, they were dissolved in a DCM/TFA 1:1 mixture (2.5 mL) at 0 °C under stirring, warmed up to room temperature, and stirred for 16 h. After removing all volatiles, the crude was dissolved in methanol and precipitated from ice-cold diethyl ether.

### Kinetic Studies

Kinetic experiments were carried out inside an argon-filled glovebox. Here is an example of the polymerization of OxP_Bn_ (M) using DBU (C1) and TU (C2) as catalysts and DiOH (I) as initiator with the following parameters: M/C1/C2/I ratio = 30/6/6/1; DCM (*V* = mL); [M] = 1 M; RT. As for the general polymerization method, the catalysts, initiator, one part of the solvent, and 30 μL of THF were mixed in a first vial and stirred for 10 min. In a separate vial, the monomer was solubilized with the other part of the solvent. The initiator/catalyst solution was then added to the vial containing the monomer. The mixture was stirred for 120 min, and aliquots of the reaction were taken at different time intervals. The aliquots were quenched with benzoic acid and used for further analysis.

### Samples Preparation for Self-Assembly

Samples for dynamic light scattering (DLS), transmission electron microscopy (TEM), and liquid atomic force microscopy (AFM) analyses were prepared in Milli-Q water at a polymer concentration of 0.1 mg/mL for P­(OxP_Me_)-*b*-P­(OxP_Bn_)-*b*-P­(OxP_Me_) copolymers and 0.25 mg/mL for P­(OxP_NH2_^+^)-*b*-P­(OxP_Bn_)-*b*-P­(OxP_NH2_^+^) copolymers. A predetermined amount of Milli-Q water was added to a block copolymer sample, and the mixture was sonicated for a maximum of 15 min. After a homogeneous solution was obtained, the mixture was magnetically stirred at 900 rpm overnight at room temperature. Samples were not filtered for analysis.

### Hydrogel Preparation

A copolymer stock solution in DMSO was prepared and transferred to several 1.5 mL vials. The samples were then lyophilized to form a polymer film. A predetermined amount of Milli-Q water was then added to the vials to prepare hydrogels with different wt %. The samples were sonicated for 15 min and placed in a thermoshaker to equilibrate overnight at room temperature. After equilibration, gelation was visually examined first using the inverted vial method.

### Fungicide Experiment

Hydrogels, with and without the fungicide (dithianon), were prepared in well plates according to the above-described procedure. For the hydrogels containing the fungicide, both copolymer and dithianon stock solutions in DMSO were prepared, transferred, and mixed in well plates. The samples were then lyophilized, resulting in the formation of a polymer film containing 30 μg of fungicide (1.5 wt/wt % of fungicide relative to the block copolymer). A predetermined amount of Milli-Q water was then added to the well plates to prepare 10 wt/vol % hydrogels. The samples were sonicated for 15 min and placed in a thermoshaker to equilibrate overnight.

In a sterile safety cabinet, a fungal spore solution was prepared from 10 days previously inoculated YMG/2 agar plates (2 g of yeast powder, 5 g of glucose, and 5 g of malt extract per liter). The spores were harvested by adding 10 mL of YMG/2 liquid medium to the plate and scraping the spores from the mycelium. The mixture was filtered using miracloth and a solution of 2000 spores per milliliter (*Phaeomoniella chlamydosporum*. CBS 101359) was adjusted by utilizing a Neubauer counting chamber. Following hydrogel formation, 0.2 mL of the fungus solution (12 μg of fungicide per mL of solution) was dispensed onto the hydrogels. The fungus solution was also dispensed onto two control experiments prepared without hydrogels. Control 1 contained only the fungicide (12 μg/mL), and control 2 was performed in the absence of any compound. The spore proliferation was evaluated using microscopy, and an optical density was measured at 600 nm (in a BioRad Benchreader). Cell density ranged between 0.15 and 1.6, indicating the absence or presence of spore growth, respectively. The microscopic evaluation was necessary since cell debris and hydrogel/dithianon mixtures strongly influenced the OD600 measurements.

### Characterization

Monomer conversions, molar masses, and block copolymer compositions were assessed by liquid-state nuclear magnetic resonance (NMR) using a Bruker Avance II 400 and Bruker Avance III 400 spectrometer at room temperature in CDCl_3_.

The determination of the molar masses $\overset{®}{\mathrm{Mn}}$ and dispersities *Đ* by size exclusion chromatography (SEC) was performed on an Agilent 1100 Series SEC system equipped with a HEMA column set (300/100/40 Å), RI and UV (254 nm) detectors. Measurements were performed at 50 °C by using DMF containing 1 mg/mL lithium bromide as the mobile phase at a flow rate of 1 mL/min. Data were obtained using poly­(methyl methacrylate) standards.

Matrix-assisted laser desorption ionization time-of-flight mass spectrometry (MALDI-ToF-MS) measurements were also performed for polymer characterization using a Bruker autoflex maX MALDI-TOF-MS/MS. Samples were prepared in chloroform or dimethylformamide, and trans-2-(3-(4-*tert*-butylphenyl)-2-methyl-2-propenyliden)­malononitrile (DCTB) with potassium trifluoroacetate as the ionizing agent was used as a matrix.

Dynamic light scattering measurements of block copolymer samples were carried out on a Zetasizer Nano-ZS (Malvern Instruments). Analyses were performed at 25 °C, using an angle of 173° and a He–Ne laser operating at 633 nm. Each spectrum is the average of 10 runs.

The samples were also analyzed by transmission electron microscopy (TEM) using a Tecnai T12 instrument from FEI, equipped with a LaB_6_ cathode (120 kV) and a BioTWIN objective lens. A MegasSYS 1k × 1k CCD sensor was used to capture images. Freshly glow-discharged copper grids (CF300-Cu, 300 mesh) coated with a 3–4 nm carbon film from Electron Microscopy Sciences (Hatfield, USA) were employed for the analyses. 5 μL of the sample was applied to the grids and left to absorb for 1 min. Then, 5 μL of a 2 wt % uranyl acetate solution was used to negatively stain the samples for 20 s. After each step, Whatman grade 1 filter papers from GE Healthcare Biosciences (Uppsala, Sweden) were employed to remove the excess of liquid.

The surface morphology of the block copolymer samples was analyzed via AFM using Cypher S Asylum Research (Oxford Instruments). The measurements were performed in blueDrive^tm^ photothermal tapping mode with an n^+^-silicon cantilever (PPP-NCHAuD) with a tip radius of <10 nm, a spring constant of 10–130 N/m, and a cantilever resonance frequency of 230 kHz. For the measurement, a sample was drop-cast (5 μL, 0.1 or 0.25 mg/mL in Milli-Q water) on a freshly cleaved Mica surface (Ted Pella, 10 mm) and incubated for 15 min. The drying of the sample was completed by an N_2_-stream for 2 min. The morphology of the self-assembled amphiphiles adsorbed on the Mica surface was observed at room temperature with a controlled thermoelectric cooling system.

To evaluate the mechanical properties of the different hydrogels, rheology measurements were performed using a stress-controlled MCR 302e rheometer from Anton Paar (Ostfildern-Scharnhausen, Germany) with a 20 mm diameter parallel-plate measuring system on a metal plate (0.15 mm gap). The storage (*G*′) and loss modulus (*G*″) of the hydrogel were determined by oscillatory frequency sweeps conducted at 20 °C with 0.01–100 rad/s at a fixed strain of 0.1%. To evaluate the nonlinear viscoelastic properties, amplitude sweeps were measured at 20 °C with the following parameters: 0.01–100% strain at a constant angular frequency of 1 rad/s.

## Results and Discussion

### Synthesis of Poly­(amino ester) ABA Triblock Copolymers

The synthesis of ABA triblock copolymers was performed by ROP of various OxPs (Figure 1) at room temperature using the reported organocatalytic system composed of DBU/TU (C1/C2) and DiOH as a bifunctional initiator (I).

**Figure 1:**
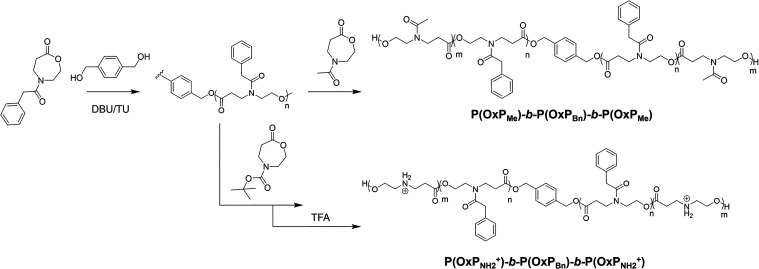
Synthetic routes of **P­(OxP_Me_)-*b*-P­(OxP_Bn_)-*b*-P­(OxP_Me_)** and **P­(OxP_NH2^+^_)-*b*-P­(OxP_Bn_)-*b*-P­(OxP_NH2^+^_)** triblock copolymers..

The controlled polymerization of OxP_Bn_ (M1) via ROP using a bifunctional initiator was first evaluated since bifunctional initiation systems have not been previously reported for OxP monomers. A kinetic study was conducted at 25 °C in dichloromethane (DCM), using the following parameters: M1/C1/C2/I ratio = 30/6/6/1; [M1] = 1 M (Figure 2). The SEC traces at different time intervals (Figure 2A) demonstrate that the polymerization yields polymers with a unimodal distribution and narrow dispersities (*Đ* < 1.13). The reaction proceeds following first-order kinetics (Figure 2B), as determined by ^1^H NMR, and shows a linear increase in the molar masses with the monomer conversion (Figure 2C). Moreover, full monomer conversion can be achieved within a reasonable reaction time (60 min), which is critical for the planned one-pot synthesis of block copolymers. MALDI analysis further demonstrates the formation of a distinct single population (Figure S4). These results confirm the controlled nature of the polymerization of the OxP_Bn_ monomer using a bifunctional initiator, thereby enabling further experiments and the synthesis of block copolymer architectures.

**Figure 2:**
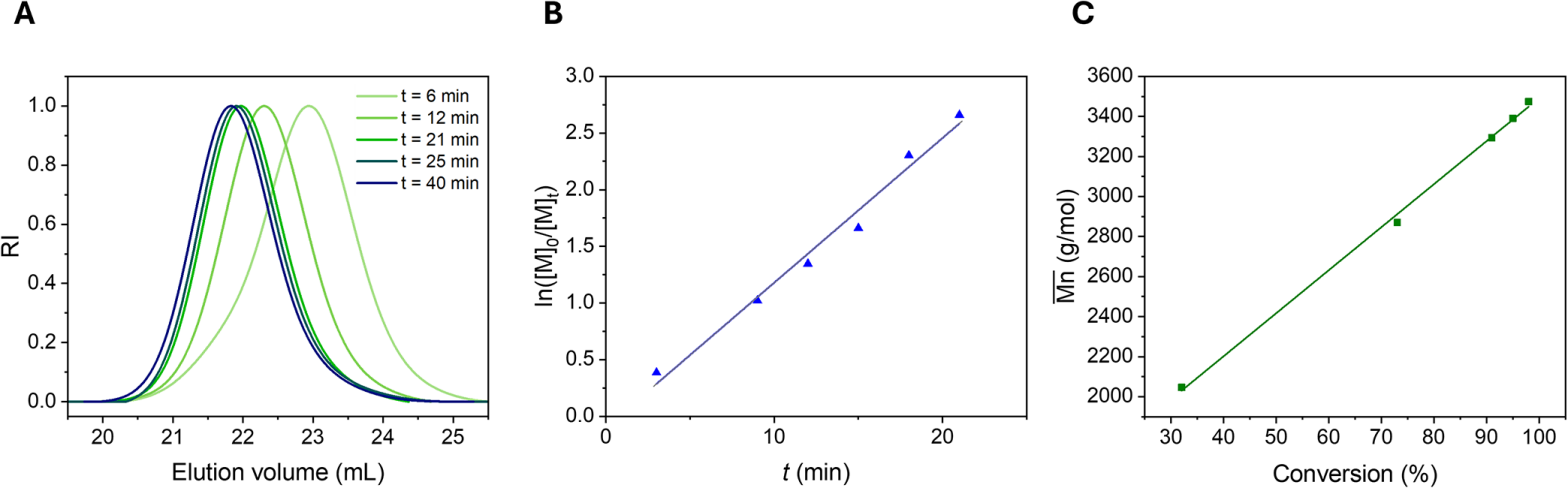
Kinetic study of the ROP of OxP_Bn_. (A) SEC elution traces at different polymerization times (RI signal, DMF, standard: PMMA). (B) ln­([M]_0_/[M]_t_) vs reaction time. (C) $\overset{®}{\mathrm{Mn}}$ vs monomer conversion.

The synthesis of P­(OxP_Me_)-*b*-P­(OxP_Bn_)-*b*-P­(OxP_Me_) and P­(OxP_NH2^+^_)-*b*-P­(OxP_Bn_)-*b*-P­(OxP_NH2^+^_) ABA triblock copolymers was performed through a one-pot copolymerization process. The reactions were conducted via sequential ROP, beginning with the OxP_Bn_ monomer followed by the OxP_Me_ or OxP_Boc_ monomers after the complete consumption of the initial monomer, as confirmed by ^1^H NMR spectroscopy.

The P­(OxP_Me_)_m_-*b*-P­(OxP_Bn_)_2n_-*b*-P­(OxP_Me_)_m_ triblock copolymers were obtained directly from consecutive ROP of the OxP_Bn_ and OxP_Me_ monomers. The conversion of both monomers was monitored through ^1^H NMR analyses (Figure S5), which also enabled the identification of the monomer repeating units in the final structural characterization of the block copolymer (Figure 3A, Table 1, run 1, **P­(OxP_Me_)_7_-*b*-P­(OxP_Bn_)_16_-*b*-P­(OxP_Me_)** **_7_**). The determination of the molar mass $\overset{®}{\mathrm{Mn}}$ _NMR_ = 6,000 g/mol of the polymer with *m* = 7 and 2*n* = 16 is consistent with the theoretical molar mass $\overset{®}{\mathrm{Mn}}$ _theo_ = 6,700 g/mol. In addition, the SEC measurements (Figure 3B) following the polymerization of OxP_Bn_ (first block, green line) and OxP_Me_ (second block, blue line) demonstrate the growth of the block copolymers with a final molar mass $\overset{®}{\mathrm{Mn}}$ _SEC_ = 6 200 g/mol and a narrow dispersity (*Đ* = 1.12).

**Figure 3:**
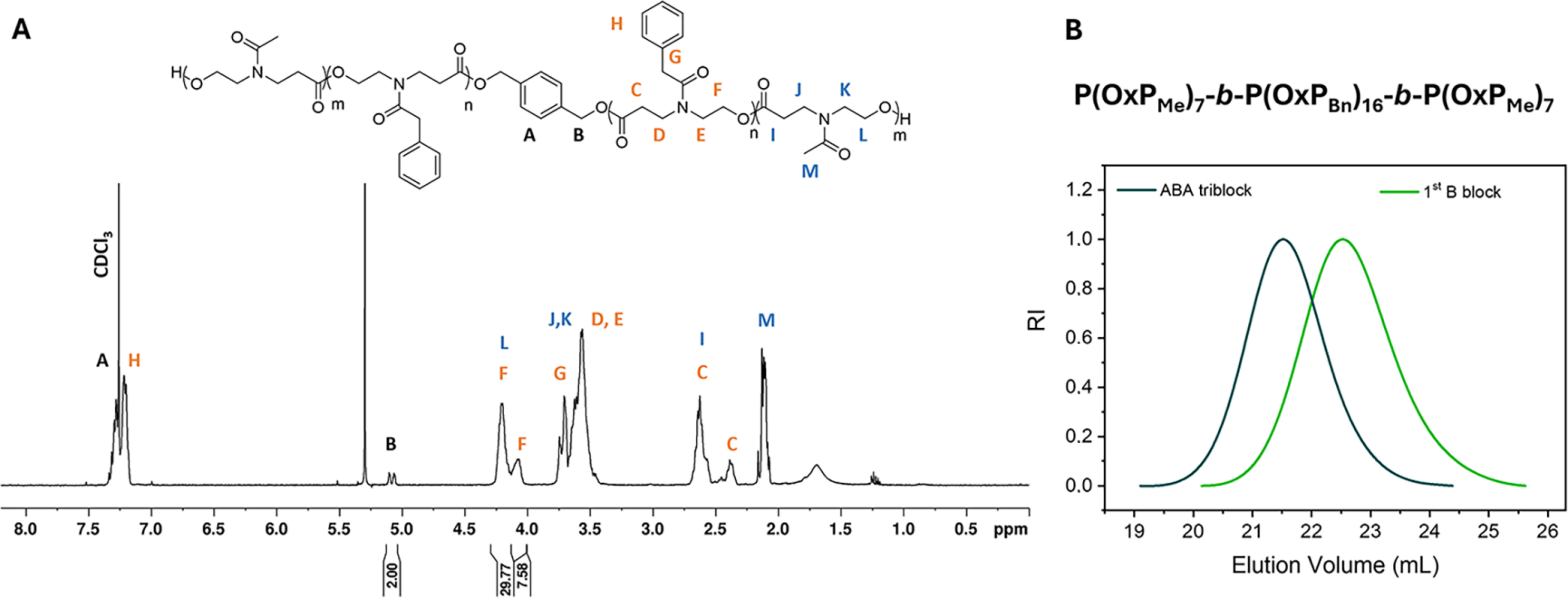
Triblock copolymer synthesis. (A) ^1^H NMR of **P­(OxP_Me_)_7_-*b*-P­(OxP_Bn_)_16_-*b*-P­(OxP_Me_)_7_** triblock copolymer in CDCl_3_ (Table 1, run 1). (B) SEC elution traces of the first block (light green) and final copolymer (dark green) (RI signal, DMF, standard: PMMA).

**Table 1:** **P­(OxP_Me_)-*b*-P­(OxP_Bn_)-*b*-P­(OxP_Me_)** and **P­(OxP_NH2^+^_)-*b*-P­(OxP_Bn_)-*b*-P­(OxP_NH2^+^_)** Triblock Copolymer Syntheses using DBU/TU as Oorganocatalysts with Different M1/M2/C1/C2/I Ratios. DCM; 25°C; [M1] = 1M; Time _first block_ = 1h; [M2] = 1M; Time _second block_ = 1.5-3h

**Run**	**M2**	M1/M2/C1/C2/I ratio	**Conv. M2 (%)** ^a^	**copolymer composition** ^a^	f1/f2 ratio^a^,^b^	$\overset{®}{Mn}$ _**theo**_(g/mol)^a^,^c^	$\overset{®}{Mn}$ _**NMR**_(g/mol)^a^,^d^	$\overset{®}{Mn}$ _**SEC**_(g/mol)^e^	***Đ*** ^e^
**1**	OxP_Me_	16/18/6/6/1	100	P(OxP_Me_)_7_-*b*-P(OxP_Bn_)_16_-*b*-P(OxP_Me_)_7_	53/47	6 700	6 000	6 200	1.12
**2**	OxP_Me_	8/18/6/6/1	100	P(OxP_Me_)_8_-*b*-P(OxP_Bn_)_8_-*b*-P(OxP_Me_)_8_	33/67	4 800	4 600	4 800	1.14
**3**	OxP_Me_	8/30/6/6/1	80	P(OxP_Me_)_14_-*b*-P(OxP_Bn_)_8_-*b*-P(OxP_Me_)_14_	22/78	6500	5 800	5 600	1.18
**4**	OxP_Me_	8/50/6/6/1	82	P(OxP_Me_)_21_-*b*-P(OxP_Bn_)_8_-*b*-P(OxP_Me_)_21_	16/84	8 400	8 500	6 500	1.21
**5**	OxP_Boc_	16/18/6/6/1	98	P(OxP_NH2_^+^)_8_-*b*-P(OxP_Bn_)_14_-*b*-P(OxP_NH2_^+^)_8_	47/53	7 600	6 300	6 700	1.12
**6**	OxP_Boc_	8/18/6/6/1	100	P(OxP_NH2_^+^)_8_-*b*-P(OxP_Bn_)_8_-*b*-P(OxP_NH2_^+^)_8_	33/67	5 700	5 700	5 100	1.13
**7**	OxP_Boc_	8/30/6/6/1	96	P(OxP_NH2_^+^)_13_-*b*-P(OxP_Bn_)_8_-*b*-P(OxP_NH2_^+^)_13_	22/78	8 300	7 600	8 100	1.12

a Calculated from ^1^H NMR. Full conversions were obtained for M1. Copolymer composition, f1/f2 ratio, $\overset{®}{\mathrm{Mn}}$ _theo_, and $\overset{®}{\mathrm{Mn}}$ _NMR_ for P­(OxP_NH2_^+^)-*b*-P­(OxP_Bn_)-*b*-P­(OxP_NH2_^+^) copolymers were determined before deprotection of P­(OxP_Boc_)-*b*-P­(OxP_Bn_)-*b*-P­(OxP_Boc_) copolymers.

b Numerical repeating unit ratio between M1 and M2 (f1/f2).

c Calculated from ^1^H NMR and feed ratio: (M.W. of BnOH) + ([M1]_0_/[BnOH]) × conv. × (M.W. of M1) + ([M2]_0_/[BnOH]) × conv. × (M.W. of M2)

d Calculated from ^1^H NMR: (M.W. of BnOH) + (DP _NMR M1_) × (M.W. of M1) + (DP _NMR M2_) × (M.W. of M2)

e Apparent molar masses obtained from SEC analysis in DMF with PMMA standards. $\overset{®}{\mathrm{Mn}}$ _SEC_ and Đ for P­(OxP_NH2_^+^)-*b*-P­(OxP_Bn_)-*b*-P­(OxP_NH2_^+^) copolymers were determined before deprotection of P­(OxP_Boc_)-*b*-P­(OxP_Bn_)-*b*-P­(OxP_Boc_) copolymers.

Following this synthetic route, a panel of P­(OxP_Me_)-*b*-P­(OxP_Bn_)-*b*-P­(OxP_Me_) triblock copolymers with various block lengths was synthesized by varying the M1/M2/C1/C2/I ratio (Table 1, runs 1–4). The block copolymers exhibit f1/f2 ratios, or hydrophobic/hydrophilic numerical repeating unit ratio, ranging from 53/47 to 16/84, as well as a variety of chain lengths $\overset{®}{\mathrm{Mn}}$ _NMR_ = 4,600–8,500 g/mol. All copolymers were obtained with controlled molar masses with values close to the theoretical values and relatively narrow dispersities (Đ ≤ 1.21). NMR characterizations (Figure S6–S8) further corroborate the synthesis of block copolymers in the presence of both OxP_Bn_ and OxP_Me_ repeating units. Finally, a single population was confirmed by ^1^H DOSY NMR spectroscopy analysis (Figure S9), and the presence of a unique diffusion coefficient.

To synthesize the polycationic P­(OxP_NH2^+^_)-*b*-P­(OxP_Bn_)-*b*-P­(OxP_NH2^+^_) triblock copolymers, the consecutive ROP of OxP_Bn_ and OxP_Boc_ monomers was first conducted in analogy to the charge-neutral P­(OxP_Me_)-*b*-P­(OxP_Bn_)-*b*-P­(OxP_Me_) triblock copolymers. As demonstrated in Figure S10, the analysis via ^1^H NMR demonstrates the conversion of both monomers. Furthermore, the SEC measurements were performed (Figure S11) show the copolymer growth following the addition of the monomers, thereby confirming the formation of a block copolymer with narrow dispersity (Đ = 1.13) (Table 1, run 6). Subsequently, the copolymer was subjected to a Boc deprotection reaction using trifluoroacetic acid (TFA) until complete removal of the protecting group was achieved (Figure S12). This procedure enabled the formation of the polycationic triblock copolymer **P­(OxP**_**NH2^+^**_ **)**_**8**_ **-** ***b*** **-P­(OxP**_**Bn**_ **)**_**8**_ **-** ***b*** **-P­(OxP**_**NH2^+^**_ **)**_**8**_. Structural characterizations of the copolymer demonstrate the presence of both OxP_Bn_ and OxP_NH2^+^_ repeating units, as evidenced by NMR (Figures S12–S14) measurements. By adjustment of the M1/M2/C1/C2/I ratio, a series of P­(OxP_NH2^+^_)-*b*-P­(OxP_Bn_)-*b*-P­(OxP_NH2^+^_) triblock copolymers were also synthesized with equivalent or similar block lengths compared to the P­(OxP_Me_)-*b*-P­(OxP_Bn_)-*b*-P­(OxP_Me_) copolymers (Table 1, runs 5–7). All polymerizations yielded copolymers with controlled molar masses and narrow dispersities (Đ ≤ 1.13).

### Self-Assembly of the Triblock Copolymers in Aqueous Media

To investigate the self-assembly properties of the amphiphilic triblock copolymers, the nanostructure formation under dilute conditions was evaluated first. Note that, due to differences in solubility, the samples were prepared in water at polymer concentrations of 0.1 mg/mL for the P­(OxP_Me_)-*b*-P­(OxP_Bn_)-*b*-P­(OxP_Me_) copolymers and 0.25 mg/mL for the P­(OxP_NH2_^+^)-*b*-P­(OxP_Bn_)-*b*-P­(OxP_NH2_^+^) copolymers. Dynamic light scattering (DLS) measurements were first conducted to determine the presence and size of the assemblies. For the P­(OxP_Me_)-*b*-P­(OxP_Bn_)-*b*-P­(OxP_Me_) copolymers, the correlogram reveals a main decay in the autocorrelation function for solutions of all triblocks iterations, with almost no sign of large-scale aggregation (Figures 4 and S15). Moreover, the intensity-weighted size distribution of each system indicates the presence of two populations above 50 nm, excluding the formation of spherical micellar structures, which are typically described by a unimodal distribution in the range of 10 to 100 nm diameter.^59^ DLS determines the hydrodynamic radius of the assemblies by assuming that their structure is spherical. However, if other structures are present, such as cylinders, two distributions can be obtained, with each mode representing the diffusion along two principal major axes, as seen in Figures 4 and S15. While nanostructure asymmetry could lead to nonaccurate hydrodynamic diameter (*D*_h_) values, qualitative differences in size distribution among the samples could be denoted. For **P­(OxP**_**Me**_ **)**_**8**_ **-** ***b*** **-P­(OxP**_**Bn**_ **)**_**8**_ **-** ***b*** **-P­(OxP**_**Me**_ **)**_**8**_, **P­(OxP**_**Me**_ **)**_**14**_ **-** ***b*** **-P­(OxP**_**Bn**_ **)**_**8**_ **-** ***b*** **-P­(OxP**_**Me**_ **)**_**14**_, and **P­(OxP**_**Me**_ **)**_**21**_ **-** ***b*** **-P­(OxP**_**Bn**_ **)**_**8**_ **-** ***b*** **-P­(OxP**_**Me**_ **)**_**21**_ copolymers, which have higher hydrophilic content, the two present intensity-weighted size distributions exhibit a different intensity contribution, a fast one and a slow one. We attribute the second one to the rotational diffusion of rod-like nanostructures along minor semiaxes. In contrast, for the **P­(OxP**_**Me**_ **)**_**7**_ **-** ***b*** **-P­(OxP**_**Bn**_ **)**_**16**_ **-** ***b*** **-P­(OxP**_**Me**_ **)**_**7**_ copolymer, the distributions appear to be overlapped and have similar intensity ratios. Thus, these results suggest that **P­(OxP**_**Me**_ **)**_**7**_ **-** ***b*** **-P­(OxP**_**Bn**_ **)**_**16**_ **-** ***b*** **-P­(OxP**_**Me**_ **)**_**7**_ could present differences in its final nanostructure compared to the copolymers discussed above.

**Figure 4:**
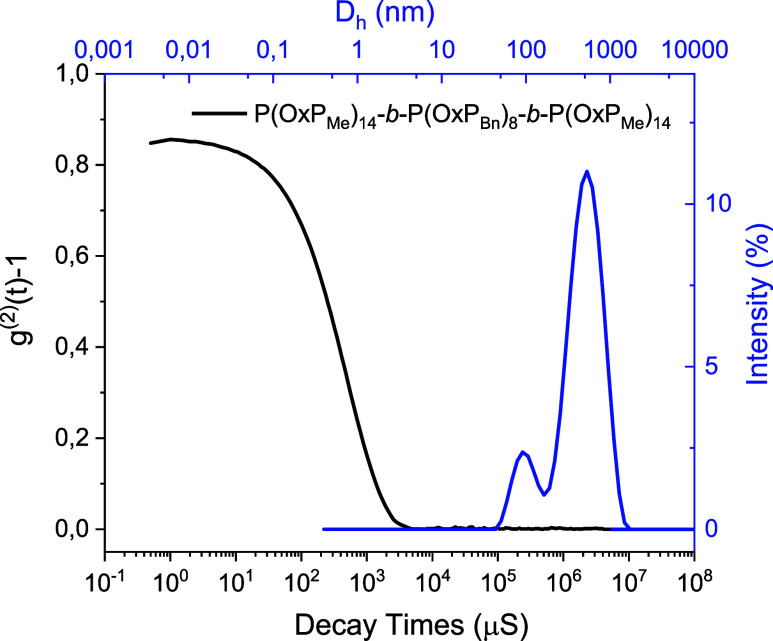
DLS correlogram (black) and intensity-weighted size distribution (blue) of **P­(OxP**_**Me**_ **)**_**14**_ **-** ***b*** **-P­(OxP**_**Bn**_ **)**_**8**_ **-** ***b*** **-P­(OxP**_**Me**_ **)**_**14**_ (Table 1, run 3) (water, 0.1 mg/mL).

To gain further insight into the nanostructures formed by the copolymers, transmission electron microscopy (TEM) was performed. The TEM images of the **P­(OxP**_**Me**_ **)**_**8**_ **-** ***b*** **-P­(OxP**_**Bn**_ **)**_**8**_ **-** ***b*** **-P­(OxP**_**Me**_ **)**_**8**_, **P­(OxP**_**Me**_ **)**_**14**_ **-** ***b*** **-P­(OxP**_**Bn**_ **)**_**8**_ **-** ***b*** **-P­(OxP**_**Me**_ **)**_**14**_, and **P­(OxP**_**Me**_ **)**_**21**_ **-** ***b*** **-P­(OxP**_**Bn**_ **)**_**8**_ **-** ***b*** **-P­(OxP**_**Me**_ **)**_**21**_ samples reveal the presence of cylindrical structures, in agreement with the two distributions observed by DLS (Figures 5A–C and S16A–C). However, differences in size can be observed, as **P­(OxP**_**Me**_ **)**_**8**_ **-** ***b*** **-P­(OxP**_**Bn**_ **)**_**8**_ **-** ***b*** **-P­(OxP**_**Me**_ **)**_**8**_ shows cylinders with a contour length of 156 ± 100 nm and 14 ± 2 nm diameter compared to the more elongated cylinders given by **P­(OxP**_**Me**_ **)**_**14**_ **-** ***b*** **-P­(OxP**_**Bn**_ **)**_**8**_ **-** ***b*** **-P­(OxP**_**Me**_ **)**_**14**_ and **P­(OxP**_**Me**_ **)**_**21**_ **-** ***b*** **-P­(OxP**_**Bn**_ **)**_**8**_ **-** ***b*** **-P­(OxP**_**Me**_ **)**_**21**_ with lengths of 372 ± 175 nm and 378 ± 122 nm, respectively (Figures S17–S22). Atomic force microscopy (AFM) measurements were also performed to characterize the self-assembly of sample **P­(OxP**_**Me**_ **)**_**7**_ **-** ***b*** **-P­(OxP**_**Bn**_ **)**_**16**_ **-** ***b*** **-P­(OxP**_**Me**_ **)**_**7**_, which could not be observed by TEM due to differences in stability during sample preparation. As shown in Figures 5D and S16D, **P­(OxP**_**Me**_ **)**_**7**_ **-** ***b*** **-P­(OxP**_**Bn**_ **)**_**16**_ **-** ***b*** **-P­(OxP**_**Me**_ **)**_**7**_ self-assembles into heterogeneous spherical aggregates or precylindrical shapes in the range of 30–85 nm, in contrast to the well-defined, anisotropic cylinders formed by the preceding samples.

**Figure 5:**
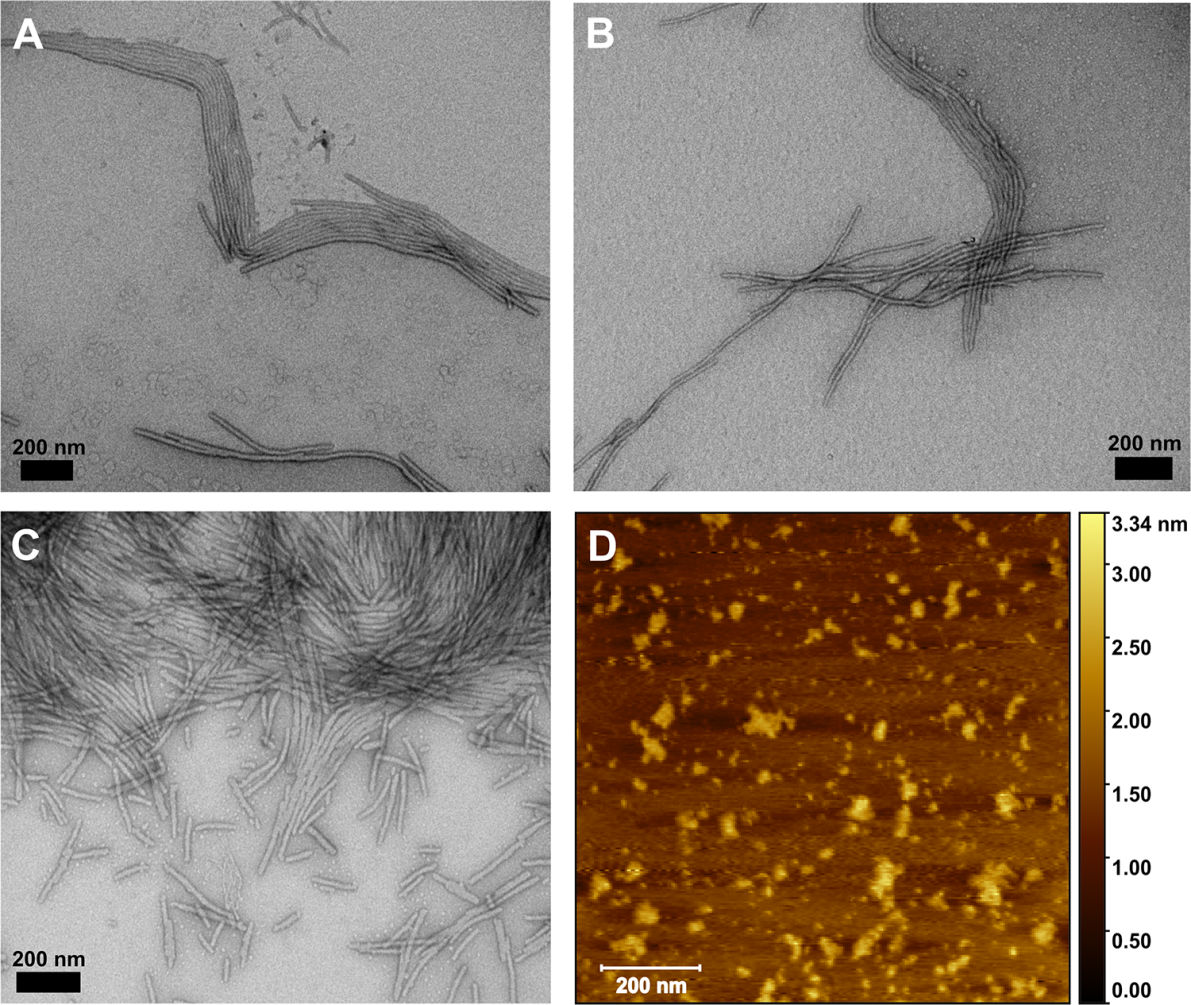
TEM and AFM images of P­(OxP_Me_)-*b*-P­(OxP_Bn_)-*b*-P­(OxP_Me_) triblock copolymers. (A) TEM images of **P­(OxP**_**Me**_ **)**_**21**_ **-** *b* **-P­(OxP**_**Bn**_ **)**_**8**_ **-** *b* **-P­(OxP**_**Me**_ **)**_**21**_. (B) TEM images of **P­(OxP**_**Me**_ **)**_**14**_ **-** *b* **-P­(OxP**_**Bn**_ **)**_**8**_ **-** *b* **-P­(OxP**_**Me**_ **)**_**14**_. (C) TEM images of **P­(OxP**_**Me**_ **)**_**8**_ **-** *b* **-P­(OxP**_**Bn**_ **)**_**8**_ **-** *b* **-P­(OxP**_**Me**_ **)**_**8**_. (D) AFM images of **P­(OxP**_**Me**_ **)**_**7**_ **-** *b* **-P­(OxP**_**Bn**_ **)**_**16**_ **-** *b* **-P­(OxP**_**Me**_ **)**_**7**_. Scale bars: 200 nm.

The diverse morphologies exhibited by the 4 samples can be explained by the structure of the block copolymers. **P­(OxP**_**Me**_ **)**_**7**_ **-** ***b*** **-P­(OxP**_**Bn**_ **)**_**16**_ **-** ***b*** **-P­(OxP**_**Me**_ **)**_**7**_, which has the highest f1/f2 ratio (Table 1), provides an insufficient hydrophilic corona to cover the hydrophobic core of the nanostructures, resulting in limited steric repulsion between the structures.^60^ This allows for core–core aggregation, leading to the formation of spherical nanoparticles or large, poorly defined aggregates. Note that the ester functionalities in the polymer backbone are classified as “hydroneutral” groups, which are neither hydrophilic nor hydrophobic. These tune down the hydrophobicity of the core, but do not balance out the interactions of the apolar backbone or the hydrophobic desolvation.^61^ By increasing the length of the hydrophilic block, the self-assembly of the copolymers shifts toward the formation of cylinders (Table 1, runs 2–4). Several studies by Luxenhofer and co-workers have reported cylindrical shapes with triblock copolymers based on poly­(oxazoline) and poly­(oxazine), which contain acetyl and phenyl pendant groups.^62,63^ They support the final cylindrical self-assembly through π–π stacking and supramolecular interactions between methyl side chain or carbonyl and aromatic group, which they describe as “sticky” aromatic groups.^62^ Interestingly, in the present work, the presence of ester bonds in the backbone of PAEs does not appear to affect the final self-assembly due to their hydroneutrality. Moreover, an additional study on amphiphilic diblock copolymers based on poly­(oxazoline) revealed a similar trend upon modulation of the length of the blocks.^64^ The copolymer with the shortest hydrophilic block resulted in spherical nanostructures that could be characterized as vesicles, which evolved into worms by increasing the length of the hydrophilic block, a trend that corroborates our experimental data.

In the case of P­(OxP_NH2_^+^)-*b*-P­(OxP_Bn_)-*b*-P­(OxP_NH2_^+^) block copolymers, DLS analyses show a similar trend, characterized by the presence of two different populations (Figure S15). AFM images (Figures 6 and S23) reveal a similar behavior compared to that of P­(OxP_Me_)-*b*-P­(OxP_Bn_)-*b*-P­(OxP_Me_) copolymers. Sample **P­(OxP**_**NH2**_^**+**^ **)**_**13**_ **-** ***b*** **-P­(OxP**_**Bn**_ **)**_**8**_ **-** ***b*** **-P­(OxP**_**NH2**_^**+**^ **)**_**13**_ with the longest hydrophilic block shows the presence of cylinders (Figure 6A) with a length and diameter of 220 ± 100 nm and 47 ± 9 nm, respectively, whereas **P­(OxP**_**NH2**_^**+**^ **)**_**8**_ **-** ***b*** **-P­(OxP**_**Bn**_ **)**_**8**_ **-** ***b*** **-P­(OxP**_**NH2**_^**+**^ **)**_**8**_ and **P­(OxP**_**NH2**_^**+**^ **)**_**8**_ **-** ***b*** **-P­(OxP**_**Bn**_ **)**_**14**_ **-** ***b*** **-P­(OxP**_**NH2**_^**+**^ **)**_**8**_ form spherical nanostructures (Figure 6B,C, respectively) with diameters of 187 ± 34 nm and 77 ± 13 nm (Figures S24–S27). However, the hydrophilic groups have a clear effect in the final assembly of the copolymers, as for an identical f1/f2 ratio, cylindrical assemblies are obtained for P­(OxP_Me_)-*b*-P­(OxP_Bn_)-*b*-P­(OxP_Me_), whereas P­(OxP_NH2_^+^)-*b*-P­(OxP_Bn_)-*b*-P­(OxP_NH2_^+^) leads to spherical objects (**P­(OxP**_**Me**_ **)**_**8**_ **-** ***b*** **-P­(OxP**_**Bn**_ **)**_**8**_ **-** ***b*** **-P­(OxP**_**Me**_ **)**_**8**_ vs **P­(OxP**_**NH2**_^**+**^ **)**_**8**_ **-** ***b*** **-P­(OxP**_**Bn**_ **)**_**8**_ **-** ***b*** **-P­(OxP**_**NH2**_^**+**^ **)**_**8**_). The presence of charges within block copolymers has been shown to enhance electrostatic repulsions between particles, thereby increasing the stability of the resulting spherical aggregates.^65^ Therefore, a longer hydrophilic block is required compared to the P­(OxP_Me_)-*b*-P­(OxP_Bn_)-*b*-P­(OxP_Me_) block copolymers to shift the transition toward the formation of cylinders.

**Figure 6:**
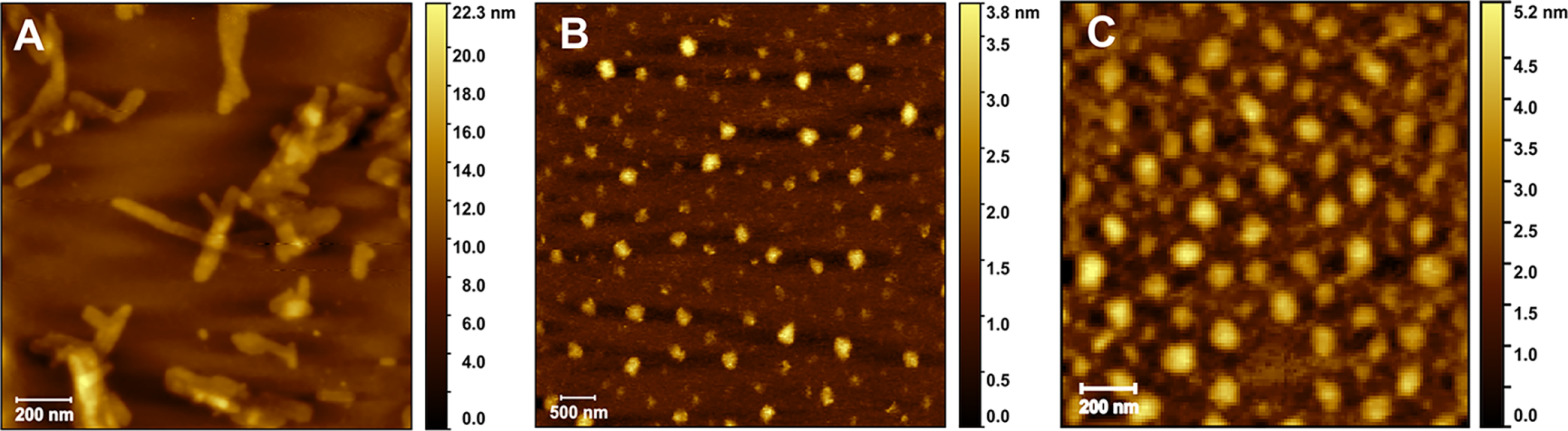
AFM images of P­(OxP_NH2_^+^)-*b*-P­(OxP_Bn_)-*b*-P­(OxP_NH2_^+^) triblock copolymers. (A) **P­(OxP**_**NH2**_^**+**^ **)**_**13**_ **-** ***b*** **-P­(OxP**_**Bn**_ **)**_**8**_ **-** ***b*** **-P­(OxP**_**NH2**_^**+**^ **)**_**13**_. (B) **P­(OxP**_**NH2**_^**+**^ **)**_**8**_ **-** *b* **-P­(OxP**_**Bn**_ **)**_**8**_ **-** ***b*** **-P­(OxP**_**NH2**_^**+**^ **)**_**8**_. (C) **P­(OxP**_**NH2**_^**+**^ **)**_**8**_ **-** ***b*** **-P­(OxP**_**Bn**_ **)**_**14**_ **-** ***b*** **-P­(OxP**_**NH2**_^**+**^ **)**_**8**_. Scale bars: 500 nm.

### Hydrogel Formation Using the Triblock Copolymers

Following the experiments focused on nanostructure formation in dilute conditions, which provided further insight into the mechanism of self-assembly, the hydrogelation properties of these ABA triblock copolymers were evaluated in a concentrated aqueous medium at 25 °C using copolymer concentrations ranging from 1.25 to 20 wt/vol %. In the series of P­(OxP_Me_)-*b*-P­(OxP_Bn_)-*b*-P­(OxP_Me_) copolymers, the **P­(OxP**_**Me**_ **)**_**7**_ **-** ***b*** **-P­(OxP**_**Bn**_ **)**_**16**_ **-** ***b*** **-P­(OxP**_**Me**_ **)**_**7**_ copolymer leads to the formation of a “heterogeneous mixture” in water, characterized by the presence of an immiscible solid/precipitate in water. This is due to the low copolymer solubility, which prevents the formation of hydrogels for all the different concentrations evaluated. **P­(OxP**_**Me**_ **)**_**8**_ **-** ***b*** **-P­(OxP**_**Bn**_ **)**_**8**_ **-** ***b*** **-P­(OxP**_**Me**_ **)**_**8**_ and **P­(OxP**_**Me**_ **)**_**14**_ **-** ***b*** **-P­(OxP**_**Bn**_ **)**_**8**_ **-** ***b*** **-P­(OxP**_**Me**_ **)**_**14**_ result in the formation of opaque homogeneous solutions, with an increase in viscosity from 1.25 to 5 wt/vol %. As shown in Figure 7 for **P­(OxP**_**Me**_ **)**_**8**_ **-** ***b*** **-P­(OxP**_**Bn**_ **)**_**8**_ **-** ***b*** **-P­(OxP**_**Me**_ **)**_**8**_, free-standing hydrogels are obtained for both copolymers at concentrations above 10 wt/vol %. The copolymer **P­(OxP**_**Me**_ **)**_**21**_ **-** ***b*** **-P­(OxP**_**Bn**_ **)**_**8**_ **-** ***b*** **-P­(OxP**_**Me**_ **)**_**21**_, which contains an elevated hydrophilic content, led to the formation of opaque homogeneous solutions with increasing viscosity at higher concentrations.

**Figure 7:**
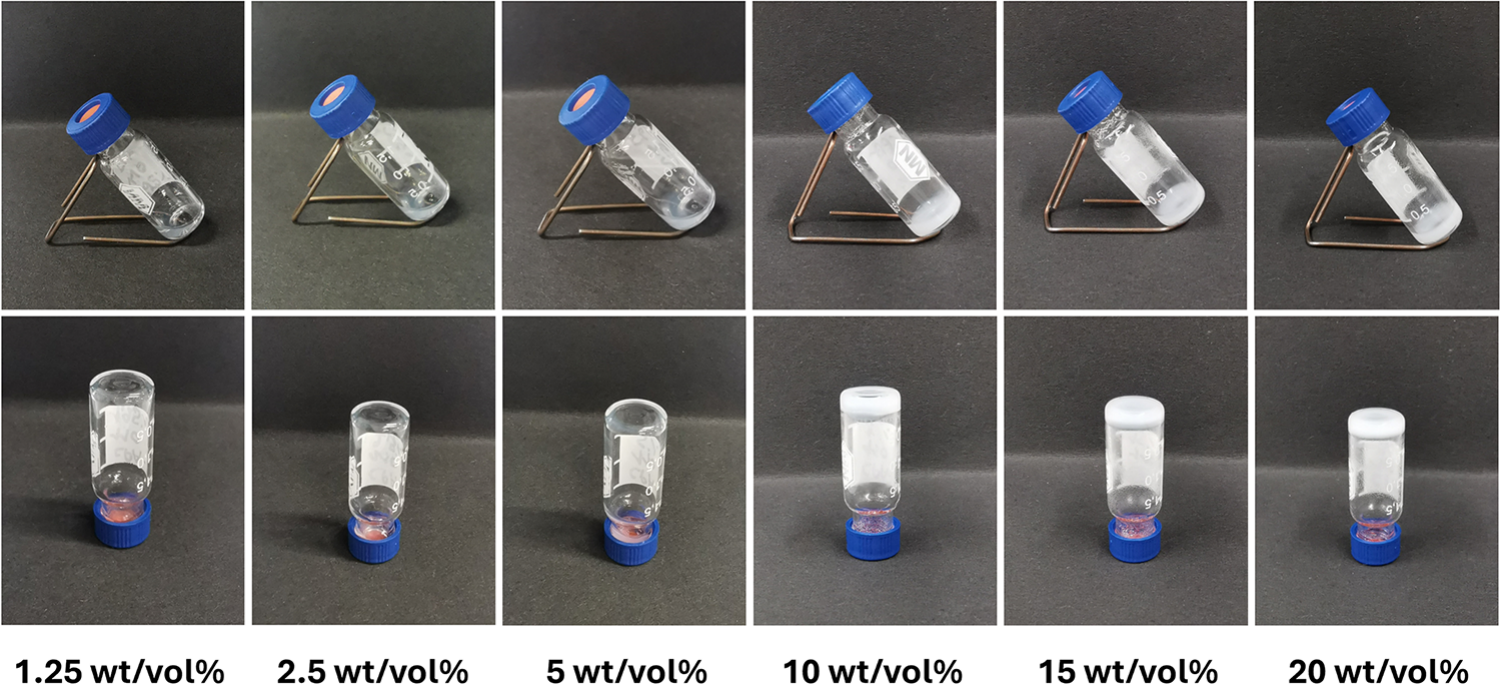
Inverted vial tests for **P­(OxP**_**Me**_ **)**_**8**_ **-** ***b*** **-P­(OxP**_**Bn**_ **)**_**8**_ **-** ***b*** **-P­(OxP**_**Me**_ **)**_**8**_ solutions at varying copolymer concentrations (water).

For P­(OxP_NH2_^+^)-*b*-P­(OxP_Bn_)-*b*-P­(OxP_NH2_^+^) copolymers, a “heterogeneous mixture” is obtained using **P­(OxP**_**NH2**_^**+**^ **)**_**8**_ **-** ***b*** **-P­(OxP**_**Bn**_ **)**_**14**_ **-** ***b*** **-P­(OxP**_**NH2**_^**+**^ **)**_**8**_ in a concentration window of 1.25 to 10 wt/vol %. However, free-standing hydrogels are formed at 15 and 20 wt/vol %, exhibiting heterogeneity due to a rapid hydrogelation of the system. Further analysis could not be performed for **P­(OxP**_**NH2**_^**+**^ **)**_**8**_ **-** ***b*** **-P­(OxP**_**Bn**_ **)**_**14**_ **-** ***b*** **-P­(OxP**_**NH2**_^**+**^ **)**_**8**_ due to the heterogeneous nature of the hydrogels. In the case of **P­(OxP**_**NH2**_^**+**^ **)**_**8**_ **-** ***b*** **-P­(OxP**_**Bn**_ **)**_**8**_ **-** ***b*** **-P­(OxP**_**NH2**_^**+**^ **)**_**8**_ copolymer, which contains longer hydrophilic blocks compared to **P­(OxP**_**NH2**_^**+**^ **)**_**8**_ **-** ***b*** **-P­(OxP**_**Bn**_ **)**_**14**_ **-** ***b*** **-P­(OxP**_**NH2**_^**+**^ **)**_**8**_, opaque homogeneous solutions are formed with increasing viscosity at higher concentrations. Finally, the **P­(OxP**_**NH2**_^**+**^ **)**_**13**_ **-** ***b*** **-P­(OxP**_**Bn**_ **)**_**8**_ **-** ***b*** **-P­(OxP**_**NH2**_^**+**^ **)**_**13**_ copolymer results in “heterogeneous mixtures”, preventing hydrogel formation.

The mechanical properties of the hydrogels were evaluated by oscillatory shear rheological experiments (Figures 8 and S28). Figure 8A shows frequency sweeps measurements of copolymer solutions using **P­(OxP**_**Me**_ **)**_**8**_ **-** ***b*** **-P­(OxP**_**Bn**_ **)**_**8**_ **-** ***b*** **-P­(OxP**_**Me**_ **)**_**8**_, **P­(OxP**_**Me**_ **)**_**14**_ **-** ***b*** **-P­(OxP**_**Bn**_ **)**_**8**_ **-** ***b*** **-P­(OxP**_**Me**_ **)**_**14**_, **P­(OxP**_**Me**_ **)**_**21**_ **-** ***b*** **-P­(OxP**_**Bn**_ **)**_**8**_ **-** ***b*** **-P­(OxP**_**Me**_ **)**_**21**_, and **P­(OxP**_**NH2**_^**+**^ **)**_**8**_ **-** ***b*** **-P­(OxP**_**Bn**_ **)**_**8**_ **-** ***b*** **-P­(OxP**_**NH2**_^**+**^ **)**_**8**_ at 10 wt/vol %. The samples are characterized by a larger storage modulus (*G*′) than the loss modulus (*G*″), indicative of a physically cross-linked network of self-assembled nanostructures^66^ for all triblock copolymer samples tested. A crossover and sol–gel transition is observed at about 2 rad/s for **P­(OxP**_**Me**_ **)**_**21**_ **-** ***b*** **-P­(OxP**_**Bn**_ **)**_**8**_ **-** ***b*** **-P­(OxP**_**Me**_ **)**_**21**_, while all other copolymer samples demonstrate high stability over the whole frequency regime. Furthermore, differences in hydrogel strength are observed among the samples, with stiffer hydrogels associated with the more hydrophobic **P­(OxP**_**Me**_ **)**_**8**_ **-** ***b*** **-P­(OxP**_**Bn**_ **)**_**8**_ **-** ***b*** **-P­(OxP**_**Me**_ **)**_**8**_ and **P­(OxP**_**Me**_ **)**_**14**_ **-** ***b*** **-P­(OxP**_**Bn**_ **)**_**8**_ **-** ***b*** **-P­(OxP**_**Me**_ **)**_**14**_ copolymers, and intermediate stiffness for **P­(OxP**_**NH2**_^**+**^ **)**_**8**_ **-** ***b*** **-P­(OxP**_**Bn**_ **)**_**8**_ **-** ***b*** **-P­(OxP**_**NH2**_^**+**^ **)**_**8**_ and the least stiff hydrogel for **P­(OxP**_**Me**_ **)**_**21**_ **-** ***b*** **-P­(OxP**_**Bn**_ **)**_**8**_ **-** ***b*** **-P­(OxP**_**Me**_ **)**_**21**_.

**Figure 8:**
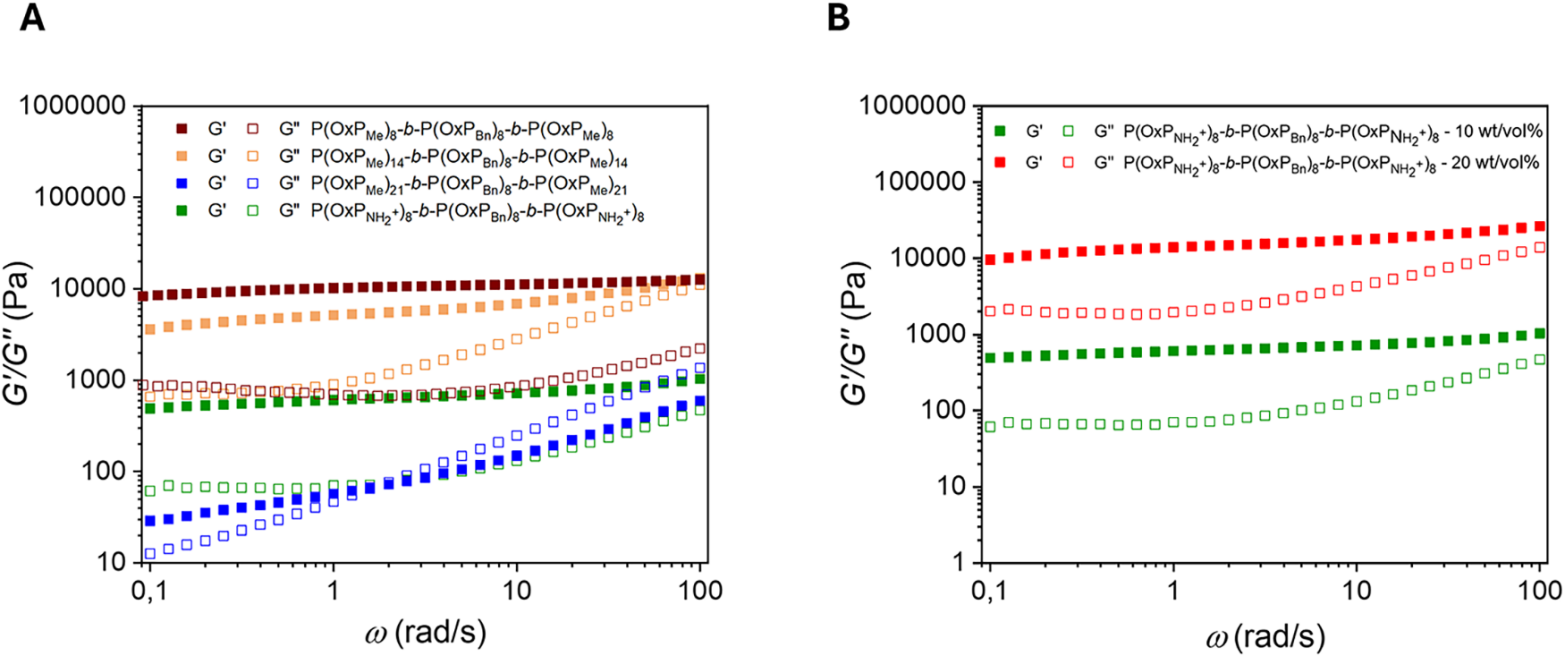
Frequency sweeps of the triblock copolymer hydrogels. (A) 10 wt/vol % hydrogels using **P­(OxP_Me_)_8_-*b*-** **P­(OxP_Bn_)_8_-*b*-P­(OxP_Me_)_8_**, **P­(OxP_Me_)_14_-*b*-P­(OxP_Bn_)_8_-*b*-P­(OxP_Me_)_14_**, **P­(OxP_Me_)_21_-*b*-P­(OxP_Bn_)_8_-*b*-P­(OxP_Me_)_21_**, and **P­(OxP_NH2_^+^)_8_-*b*-P­(OxP_Bn_)_8_-*b*-P­(OxP_NH2_^+^)**_**8**_ (water). (B) Comparison between 10 wt/vol % (green) and 20 wt/vol % (red) hydrogels using **P­(OxP**_**NH2**_^**+**^ **)**_**8**_ **-** ***b*** **-P­(OxP**_**Bn**_ **)**_**8**_ **-** ***b*-** **P­(OxP**_**NH2**_^**+**^ **)**_**8**_ (water).

In accordance with the results by Luxenhofer and colleagues,^62^ involving comparable triblock polyoxazoline and polyoxazine copolymers, we observed that P­(OxP_Me_)-*b*-P­(OxP_Bn_)-*b*-P­(OxP_Me_) copolymers, which self-assemble into cylindrical shapes in dilute solution (Table 1, runs 2–4), also form hydrogels at higher copolymer concentrations. This finding was supported by shear rheology, confirming a strong driving force for hydrogelation.

The difference in gel strength among the P­(OxP_Me_)-*b*-P­(OxP_Bn_)-*b*-P­(OxP_Me_) samples can be related to their macromolecular composition. **P­(OxP**_**Me**_ **)**_**21**_ **-** ***b*** **-P­(OxP**_**Bn**_ **)**_**8**_ **-** ***b*** **-P­(OxP**_**Me**_ **)**_**21**_ exhibits a higher degree of hydrophilicity compared to **P­(OxP**_**Me**_ **)**_**8**_ **-** ***b*** **-P­(OxP**_**Bn**_ **)**_**8**_ **-** ***b*** **-P­(OxP**_**Me**_ **)**_**8**_ and **P­(OxP**_**Me**_ **)**_**14**_ **-** ***b*** **-P­(OxP**_**Bn**_ **)**_**8**_ **-** ***b*** **-P­(OxP**_**Me**_ **)**_**14**_, owing to its extended hydrophilic block, which can prevent efficient intercylinder interactions and aggregation due to higher steric repulsions and, subsequently, hydrogelation. This results in a decrease in the hydrogel stability and could potentially lead to the G’/G’’ crossover, as shown in Figure 8A. **P­(OxP**_**Me**_ **)**_**21**_ **-** ***b*** **-P­(OxP**_**Bn**_ **)**_**8**_ **-** ***b*** **-P­(OxP**_**Me**_ **)**_**21**_ is stable at low deformation frequencies but shows a transition from a hydrogel with solid-like properties to a gel featuring viscous flow. Compared to the other copolymer hydrogel compositions, this indicates a more dynamic or weakly structured network with a high viscous component that cannot maintain its stability under rapid deformation. The crossover at ω ≈ 2 rad/s corresponds to a relaxation time τ *=* 0.5 s, suggesting that the hydrogel’s network is dynamically maintained by reversible cross-links.

For the P­(OxP_NH2_^+^)-*b*-P­(OxP_Bn_)-*b*-P­(OxP_NH2_^+^) series, hydrogels using **P­(OxP**_**NH2**_^**+**^ **)**_**8**_ **-** ***b*** **-P­(OxP**_**Bn**_ **)**_**14**_ **-** ***b*** **-P­(OxP**_**NH2**_^**+**^ **)**_**8**_ and **P­(OxP**_**NH2**_^**+**^ **)**_**8**_ **-** ***b*** **-P­(OxP**_**Bn**_ **)**_**8**_ **-** ***b*** **-P­(OxP**_**NH2**_^**+**^ **)**_**8**_ copolymers were also obtained. **P­(OxP**_**NH2**_^**+**^ **)**_**8**_ **-** ***b*** **-P­(OxP**_**Bn**_ **)**_**14**_ **-** ***b*** **-P­(OxP**_**NH2**_^**+**^ **)**_**8**_ triblock copolymers exhibit a more pronounced hydrophobicity compared to **P­(OxP**_**NH2**_^**+**^ **)**_**8**_ **-** ***b*** **-P­(OxP**_**Bn**_ **)**_**8**_ **-** ***b*** **-P­(OxP**_**NH2**_^**+**^ **)**_**8**_ and **P­(OxP**_**NH2**_^**+**^ **)**_**13**_ **-** ***b*** **-P­(OxP**_**Bn**_ **)**_**8**_ **-** ***b*** **-P­(OxP**_**NH2**_^**+**^ **)**_**13**_, resulting in the formation of a dense and rigid core. Furthermore, the hydrophilic block potentially enables bridging between particles and the formation of hydrogen bonds from ammonium functionalities, leading to percolation and hydrogelation of the system. The mechanical properties of 10 and 20 wt/vol % hydrogels using **P­(OxP**_**NH2**_^**+**^ **)**_**8**_ **-** ***b*** **-P­(OxP**_**Bn**_ **)**_**8**_ **-** ***b*** **-P­(OxP**_**NH2**_^**+**^ **)**_**8**_ were also investigated (Figures 8B and S28B). As shown in Figure 8B, the 20 wt/vol % hydrogel exhibits higher gel stiffness. This increase is attributed to a higher density of polymer chains, which promotes more chain entanglements and results in a denser network structure. Additionally, the reduced water content in higher concentration gels leads to lower compressibility, which directly correlates with improved mechanical stability.^67^

Overall, these results demonstrate that the properties of hydrogels are modulated by altering the composition of the copolymers and their f1/f2 ratio, as well as the concentration of the amphiphilic ABA triblock copolymers.

### Fungicide Formulation in Triblock Copolymer-Based Hydrogels

In the final step of the study, we investigated the potential of PAE-based hydrogels for the formulation of a water-insoluble fungicide and the inhibitory effect on spore proliferation of the fungus *Phaeomoniella chlamydosporum* in view of future agrochemical and plant protection applications. Dithianon is a water-insoluble fungicide used for its ability to inhibit the mycelial growth and conidial germination in fungal organisms.^68^ Here, the antifungal properties of dithianon were evaluated while formulated in the various hydrogels described and characterized in the previous section. We focused our investigations on *Phaeomoniella chlamydosporum*, a fungus generally associated with esca disease in mature grapevines. In the presented experiments, we therefore used a fungal spore solution to determine whether the hydrogels containing the fungicide (12 μg/mL of spore solution) would enable the inhibition of spore proliferation.

Two series of experiments were conducted. The first series involved the preparation of 10 wt/vol % hydrogel using **P­(OxP**_**Me**_ **)**_**8**_ **-** ***b*** **-P­(OxP**_**Bn**_ **)**_**8**_ **-** ***b*** **-P­(OxP**_**Me**_ **)**_**8**_, **P­(OxP**_**Me**_ **)**_**14**_ **-** ***b*** **-P­(OxP**_**Bn**_ **)**_**8**_ **-** ***b*** **-P­(OxP**_**Me**_ **)**_**14**_, **P­(OxP**_**Me**_ **)**_**21**_ **-** ***b*** **-P­(OxP**_**Bn**_ **)**_**8**_ **-** ***b*** **-P­(OxP**_**Me**_ **)**_**21**_, and **P­(OxP**_**NH2**_^**+**^ **)**_**8**_ **-** ***b*** **-P­(OxP**_**Bn**_ **)**_**8**_ **-** ***b*** **-P­(OxP**_**NH2**_^**+**^ **)**_**8**_ triblock copolymers, without the incorporation of fungicide, to serve as a control experiment. The second series of hydrogels was prepared by incorporating dithianon at 1.5 wt/wt % relative to the amphiphilic block copolymers. Two control experiments were also performed, in which hydrogels were excluded. Control 1 consisted of the sole presence of the fungicide, while control 2 was performed in the absence of any compound.

As demonstrated in Table 2 and Figure S29, experiment control 1, which was conducted exclusively using the fungicide, exhibits a negligible spore proliferation with a value of 0.204. It is important to note that due to the insolubility of dithianon in water, a fungicide solution in DMSO was first disposed in the well plates, followed by drying and removal of the organic solvent, before the spore solution was finally added to the well plate to test spore proliferation. The use of organic solvents is obviously not a practical formulation in view of agrochemical and plant protection applications. In contrast, experiment control 2, conducted in the absence of any compound, allows for spore growth (1.081). In the case of **P­(OxP**_**NH2**_^**+**^ **)**_**8**_ **-** ***b*** **-P­(OxP**_**Bn**_ **)**_**8**_ **-** ***b*** **-P­(OxP**_**NH2**_^**+**^ **)**_**8**_ hydrogel, low spore proliferation is observed without or with the presence of dithianon (0.316 and 0.379, Figures 9D and 9D′ respectively), as expected for cationic block copolymers which interact and disrupt the fungal cell membrane. Therefore, these results validate the efficiency of the system, with the aim of evaluating the potential of the neutral PAE block copolymer hydrogel materials to formulate the water-insoluble dithianon and its impact on fungus proliferation. The use of **P­(OxP**_**Me**_ **)**_**8**_ **-** ***b*** **-P­(OxP**_**Bn**_ **)**_**8**_ **-** ***b*** **-P­(OxP**_**Me**_ **)**_**8**_ and **P­(OxP**_**Me**_ **)**_**14**_ **-** ***b*** **-P­(OxP**_**Bn**_ **)**_**8**_ **-** ***b*** **-P­(OxP**_**Me**_ **)**_**14**_ hydrogels without fungicide results in clear spore proliferation, with values of 1.568 and 1.264, respectively (Figure 9A,B). However, these values decrease by 50% to 1.065 and 0.869 in the presence of the fungicide (Figure 9A,B′), indicating a reduction in spore proliferation due to the activity of the fungicide. Interestingly, a much more pronounced impact on spore proliferation is apparent for the experiments using **P­(OxP**_**Me**_ **)**_**21**_ **-** ***b*** **-P­(OxP**_**Bn**_ **)**_**8**_ **-** ***b*** **-P­(OxP**_**Me**_ **)**_**21**_, with values decreased from 0.901 to 0.284 (decrease of 69%) (Figure 9C,C′).

**Table 2:**
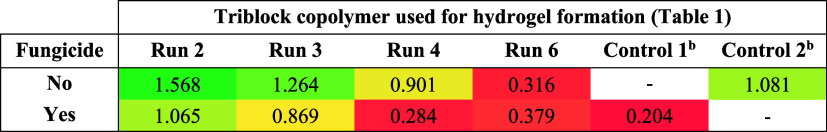
Spore Proliferation^a^ as a Function of the Different Hydrogels and Fungicides^c^

a Spore proliferation is evaluated on a scale from 0.15 to 1.6, with a color gradient ranging from red to green; typical measurement errors are ± 0.5%. Low spore growth is closer to 0.15 (red), while significant spore proliferation is closer to 1.6 (green).

b Control experiments 1 and 2 were performed without hydrogels.

c Parameters: Fungicide quantity per mL of spore solution = 12 μg/mL; Fungicide quantity relative to the triblock copolymer = 1.5 wt/wt%; triblock copolymer concentration in the hydrogel = 10 wt/vol.

**Figure 9:**
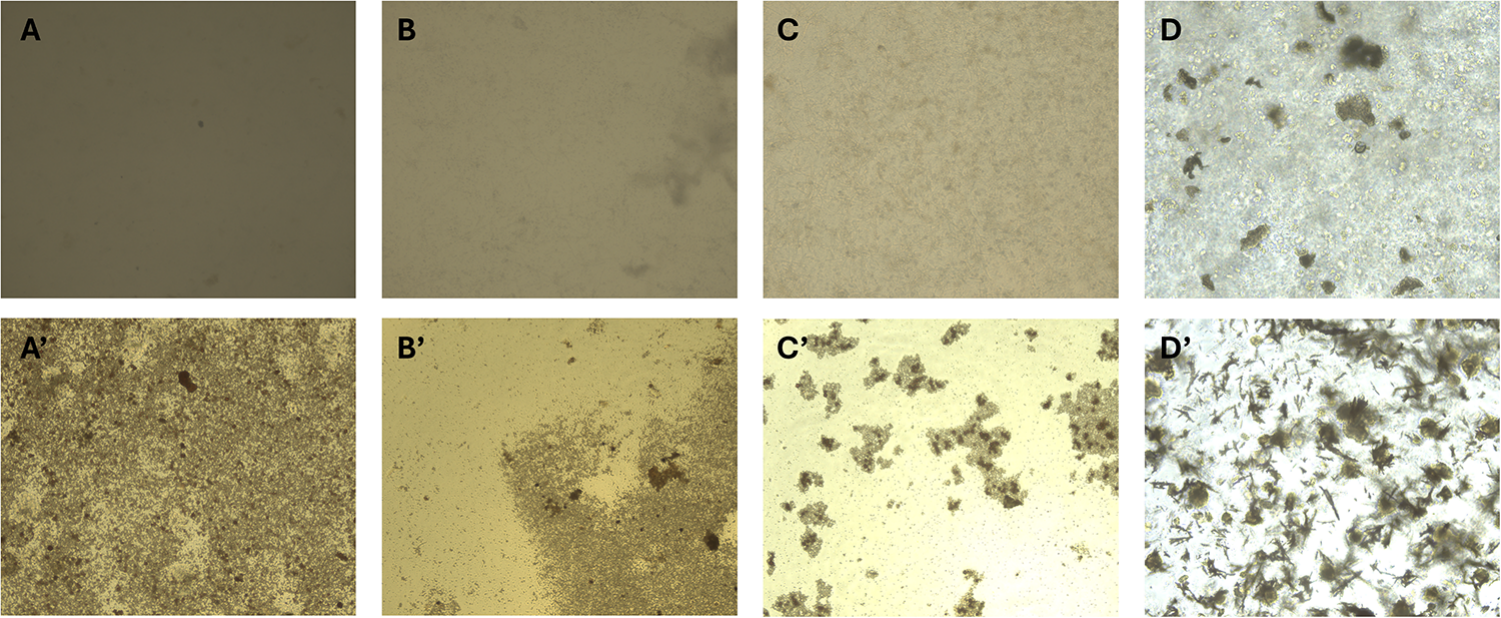
Microscopy images of the spore growth experiments. From left to right: hydrogels using **P­(OxP**_**Me**_ **)**_**8**_ **-*b*-P­(OxP**_**Bn**_ **)**_**8**_ **-*b*-P­(OxP**_**Me**_ **)**_**8**_ (A), **P­(OxP**_**Me**_ **)**_**14**_ **-*b*-P­(OxP**_**Bn**_ **)**_**8**_ **-*b*-P­(OxP**_**Me**_ **)**_**14**_ (B), **P­(OxP**_**Me**_ **)**_**21**_ **-*b*-P­(OxP**_**Bn**_ **)**_**8**_ **-*b*-P­(OxP**_**Me**_ **)**_**21**_ (C), and **P­(OxP**_**NH2**_^**+**^ **)**_**8**_ **-*b*-P­(OxP**_**Bn**_ **)**_**8**_ **-*b*-P­(OxP**_**NH2**_^**+**^ **)**_**8**_ (D) triblock copolymers in the absence of fungicide, first row. Hydrogels using **P­(OxP**_**Me**_ **)**_**8**_ **-*b*-P­(OxP**_**Bn**_ **)**_**8**_ **-*b*-P­(OxP**_**Me**_ **)**_**8**_ (A′), **P­(OxP**_**Me**_ **)**_**14**_ **-*b*-P­(OxP**_**Bn**_ **)**_**8**_ **-*b*-P­(OxP**_**Me**_ **)**_**14**_ (B′), **P­(OxP**_**Me**_ **)**_**21**_ **-*b*-P­(OxP**_**Bn**_ **)**_**8**_ **-*b*-P­(OxP**_**Me**_ **)**_**21**_ (C′), and **P­(OxP**_**NH2**_^**+**^ **)**_**8**_ **-*b*-P­(OxP**_**Bn**_ **)**_**8**_ **-*b*-P­(OxP**_**NH2**_^**+**^ **)**_**8**_ (D′) triblock copolymers in the presence of fungicide, second row.

In general, all hydrogel samples containing dithianon reduced the proliferation of spores from the fungus solution, thereby demonstrating the availability of the fungicide in time. The variation in antifungal properties among the samples most likely can be attributed to a combination of changes in the macromolecular composition of the block copolymer and, to a smaller degree, to changes in the mechanical properties of the hydrogels. As discussed previously, block copolymers **P­(OxP**_**Me**_ **)**_**8**_ **-** ***b*** **-P­(OxP**_**Bn**_ **)**_**8**_ **-** ***b*** **-P­(OxP**_**Me**_ **)**_**8**_ and **P­(OxP**_**Me**_ **)**_**14**_ **-** ***b*** **-P­(OxP**_**Bn**_ **)**_**8**_ **-** ***b*** **-P­(OxP**_**Me**_ **)**_**14**_ exhibit higher levels of hydrophobicity compared to sample **P­(OxP**_**Me**_ **)**_**21**_ **-** ***b*** **-P­(OxP**_**Bn**_ **)**_**8**_ **-** ***b*** **-P­(OxP**_**Me**_ **)**_**21**_, and further result in the formation of more rigid hydrogels within this series. In this experiment, **P­(OxP**_**Me**_ **)**_**8**_ **-** ***b*** **-P­(OxP**_**Bn**_ **)**_**8**_ **-** ***b*** **-P­(OxP**_**Me**_ **)**_**8**_ and **P­(OxP**_**Me**_ **)**_**14**_ **-** ***b*** **-P­(OxP**_**Bn**_ **)**_**8**_ **-** ***b*** **-P­(OxP**_**Me**_ **)**_**14**_ show the lowest antifungal properties among all the samples tested. In contrast, **P­(OxP**_**Me**_ **)**_**21**_ **-** ***b*** **-P­(OxP**_**Bn**_ **)**_**8**_ **-** ***b*** **-P­(OxP**_**Me**_ **)**_**21**_, exhibiting the lowest f1/f2 ratio and thus larger hydrophilic block size, afforded the most flexible hydrogels and demonstrated the strongest antifungal properties. Most likely, the flexible **P­(OxP**_**Me**_ **)**_**21**_ **-** ***b*** **-P­(OxP**_**Bn**_ **)**_**8**_ **-** ***b*** **-P­(OxP**_**Me**_ **)**_**21**_ hydrogels, with higher hydrophilic character and thereby lower loading capacity, allow for better fungicide availability, which results in an improved reduction of spore proliferation. This is corroborated by experimental results for **P­(OxP**_**Me**_ **)**_**21**_ **-** ***b*** **-P­(OxP**_**Bn**_ **)**_**8**_ **-** ***b*** **-P­(OxP**_**Me**_ **)**_**21**_ (Figure 9C′) showing debris of dead cells, while other samples do show intact mycelium. In summary, these results demonstrate the potential of the tunable PEA triblock copolymer hydrogels as a delivery platform for water-insoluble fungicide release systems and their translation as dispensable and biodegradable formulations in the agrochemical sector.

## Conclusion

A series of amphiphilic block copolymers based on poly­(β-amino ester)­s was synthesized through a one-pot ROCP of various OxP monomers at room temperature. The ABA triblock copolymers P­(OxP_Me_)-*b*-P­(OxP_Bn_)-*b*-P­(OxP_Me_) and P­(OxP_NH2_^+^)-*b*-P­(OxP_Bn_)-*b*-P­(OxP_NH2_^+^) were synthesized with controlled molar masses and narrow dispersities (Đ < 1.21) using DBU/TU organocatalysts and DiOH as an initiator. Varying the M1/M2/C1/C2 ratio afforded a panel of amphiphilic block copolymers with various compositions and hydrophobic/hydrophilic (f1/f2) ratios ranging from 53/47 to 16/84.

A study of the self-assembly of the amphiphilic block copolymers in a dilute aqueous solution was conducted to investigate the formation of nanostructures. DLS and TEM analyses demonstrated the formation of several morphologies, including cylindrical and spherical assemblies, depending on the nature and the PAE block lengths of the copolymers. In both series of polymeric materials, it was demonstrated that the more hydrophobic copolymers led to the formation of large spherical nanostructures that transitioned toward the formation of cylinders by increasing the hydrophilic block length.

Afterward, hydrogel formation in concentrated media was performed for several block copolymers and characterized by shear rheology experiments for samples **P­(OxP**_**Me**_ **)**_**8**_ **-** ***b*** **-P­(OxP**_**Bn**_ **)**_**8**_ **-** ***b*** **-P­(OxP**_**Me**_ **)**_**8**_, **P­(OxP**_**Me**_ **)**_**14**_ **-** ***b*** **-P­(OxP**_**Bn**_ **)**_**8**_ **-** ***b*** **-P­(OxP**_**Me**_ **)**_**14**_, **P­(OxP**_**Me**_ **)**_**21**_ **-** ***b*** **-P­(OxP**_**Bn**_ **)**_**8**_ **-** ***b*** **-P­(OxP**_**Me**_ **)**_**21**_, and **P­(OxP**_**NH2**_^**+**^ **)**_**8**_ **-** ***b*** **-P­(OxP**_**Bn**_ **)**_**8**_ **-** ***b*** **-P­(OxP**_**NH2**_^**+**^ **)**_**8**_. The rheological properties of the hydrogels could be modulated by altering the copolymer composition or f1/f2 ratio and copolymer concentration. Specifically, the stiffer hydrogels were obtained when increasing the hydrophobic character of the copolymers and when increasing the copolymer concentrations.

Finally, the formulation of a hydrophobic fungicide in hydrogels and its effect on the spore proliferation were tested. All samples exhibited antifungal properties, resulting in a reduction in the spore growth. However, the most flexible hydrogel based on a PAE block copolymer with the highest hydrophilic character (**P­(OxP**_**Me**_ **)**_**21**_ **-** ***b*** **-P­(OxP**_**Bn**_ **)**_**8**_ **-** ***b*** **-P­(OxP**_**Me**_ **)**_**21**_) allowed for an enhanced availability of the fungicide, leading to a more prominent decrease in spore proliferation among the samples.

The synthesis and development of the described poly­(β-amino ester) triblock copolymer-based biomaterials have revealed promising results regarding the formulation of low molecular weight apolar fungicides in view of agrochemical applications. Given the diverse composition of the copolymers, particularly the presence or absence of charges, as well as the tunable supramolecular nanostructure and mechanical properties resulting from their hydrogelation, a much wider variety of compounds can be considered, ranging from hydrophobic molecules to DNA or RNA for drug and gene delivery. These new materials provide exciting opportunities, particularly in applications as polymeric delivery systems in the agrochemical and biomedical fields.

## Supplementary Material



## Notes

(NMR spectroscopy, SEC, MALDI-TOF-MS, and DLS analysis, TEM and AFM micrographs and analysis, shear rheology analysis, spore proliferation images) (PDF)

## #

Department of Physical Chemistry and Applied Thermodynamics, University of Córdoba, Córdoba, Spain
