# Supplementary material for: Synthesis, Supramolecular Assembly, and Hydrogelation of Poly(amino ester) ABA Triblock Copolymers
Source: Biomacromolecules. 2025 Dec 15;27(1):567–79. doi: 10.1021/acs.biomac.5c01828 (PMC12801195; doi:10.1021/acs.biomac.5c01828)
Supplement: Supplementary file 1 [file bm5c01828_si_001.pdf]

Synthesis, supramolecular assembly and hydrogelation of poly(amino ester) ABA triblock copolymers

Chloé Pascouau,[‡a] Kamila Wittek,[‡a] Jessica Erlenbusch,[a] Sebastian Becker,[a] Jochen Fischer-Schuch,[b] Pablo G. Argudo,[c,d] and Pol Besenius[*a]

[a]Department of Chemistry, Johannes Gutenberg-University Mainz, Duesbergweg 10-14, D-55128 Mainz (Germany).

[b]Institut für Biotechnologie und Wirkstoff-Forschung gGmbH, Hanns-Dieter-Hüsch-Weg 17, 55128 Mainz (Germany)

[c]Department of Molecular Spectroscopy, Max Planck Institute for Polymer Research, Ackermannweg 10, 55128 Mainz (Germany)

[d]present address: Department of Physical Chemistry and Applied Thermodynamics, University of Córdoba, Córdoba, Spain

*Corresponding author E-mail address: besenius@uni-mainz.de

Figure S1 3
Figure S2 3
Figure S3 4
Figure S4 4
Figure S5 5
Figure S6 6
Figure S7 7
Figure S8 8
Figure S9 9
Figure S10 10
Figure S11 11
Figure S12 11
Figure S13 12
Figure S14 13
Figure S15 14
Figure S16 15
Figure S17 16
Figure S18 16
Figure S19 16
Figure S20 17
Figure S21 17
Figure S22 17
Figure S23 18
Figure S24 19
Figure S25 19
Figure S26 19
Figure S27 20
Figure S28 20
Figure S29 21

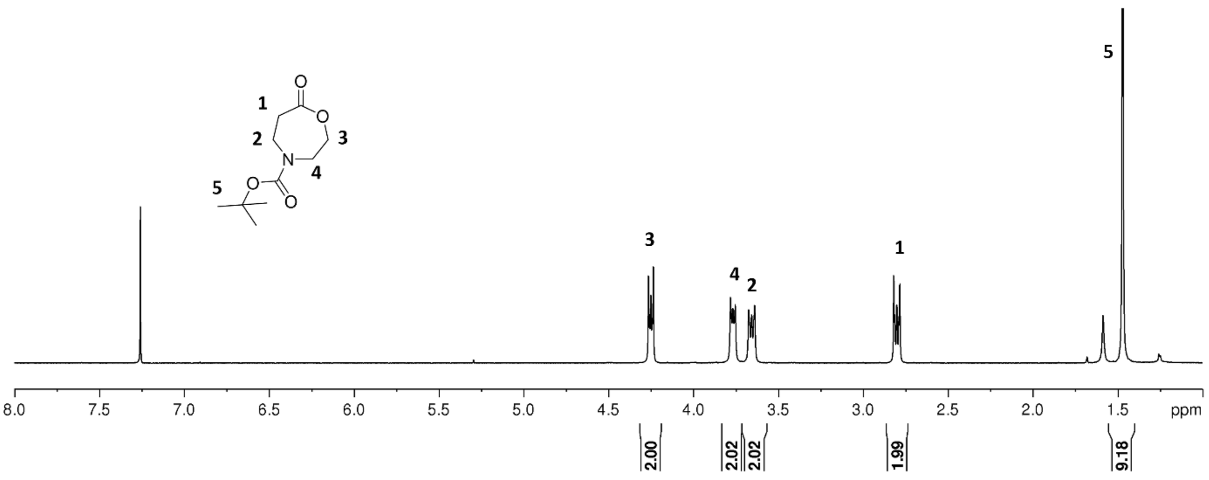

***Figure S1**: ¹H NMR of OxP_{Boc} in $CDCl_3$.*

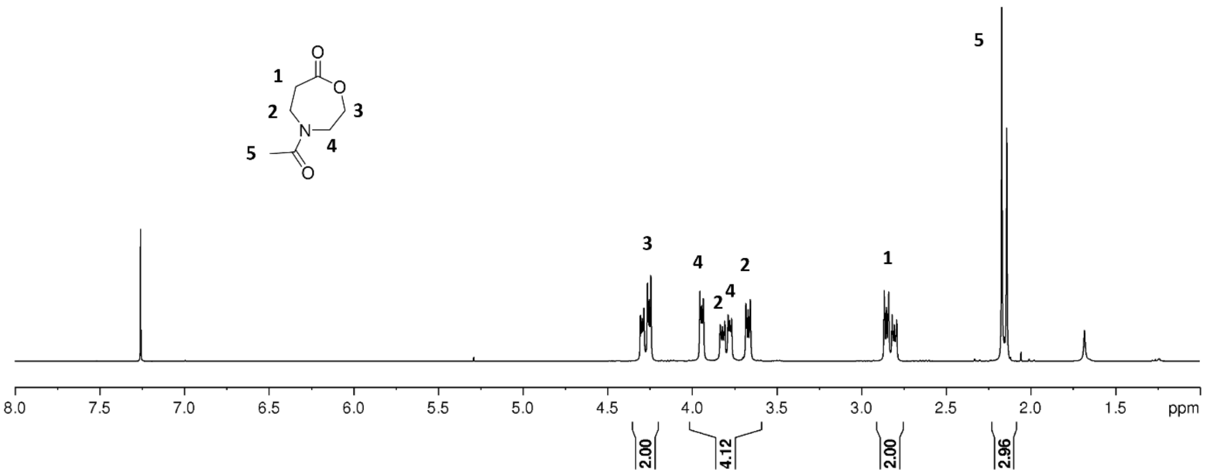

***Figure S2**: ¹H NMR of OxP_{Me} in $CDCl_3$.*

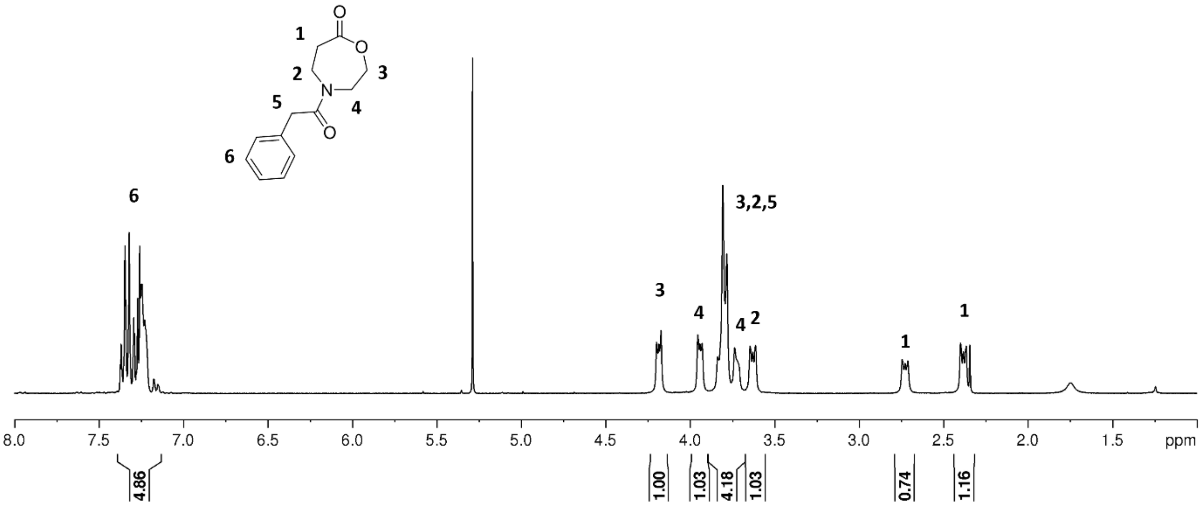

Figure S3: *^{1}H NMR of OxP$_{Bn}$ in CDCl$_3$.*

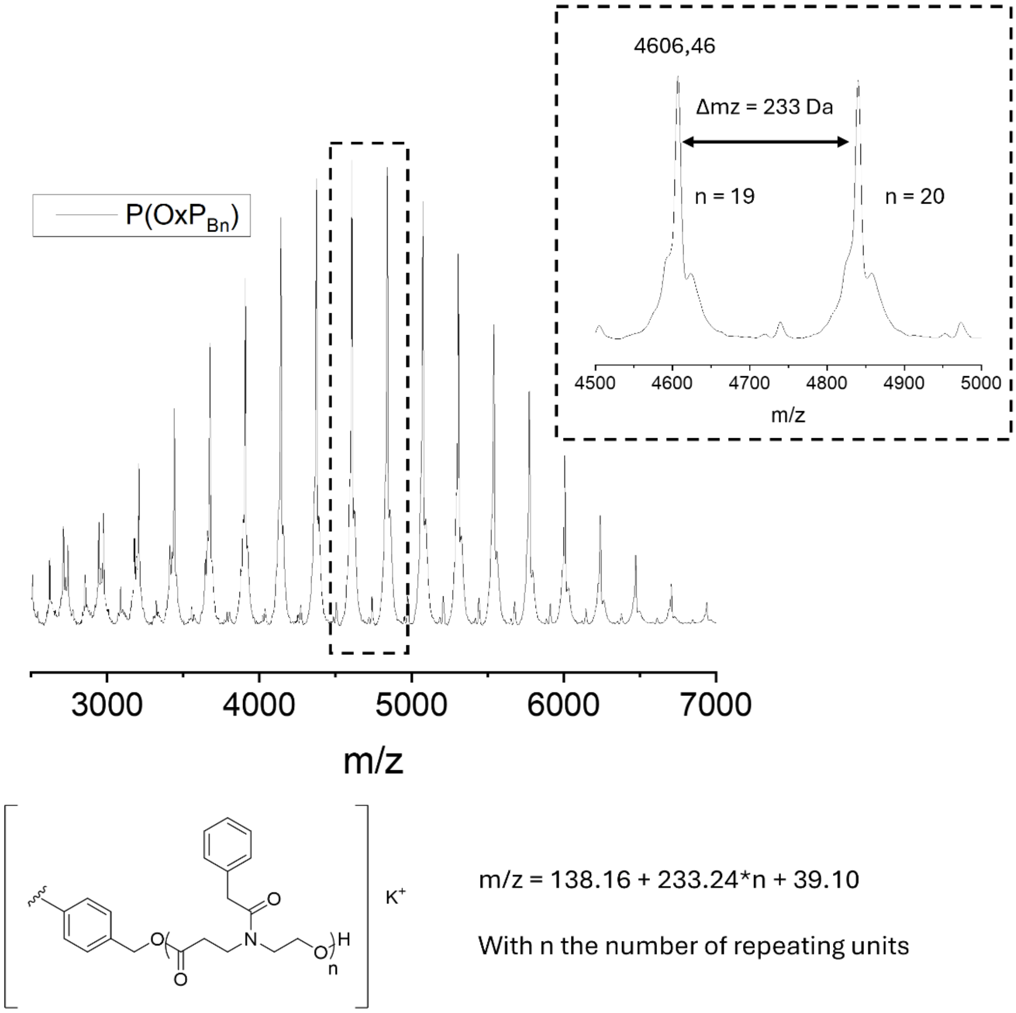

Figure S4: *MALDI-ToF mass spectrum of P(OxP$_{Bn}$) (DCTB, KTFA).*

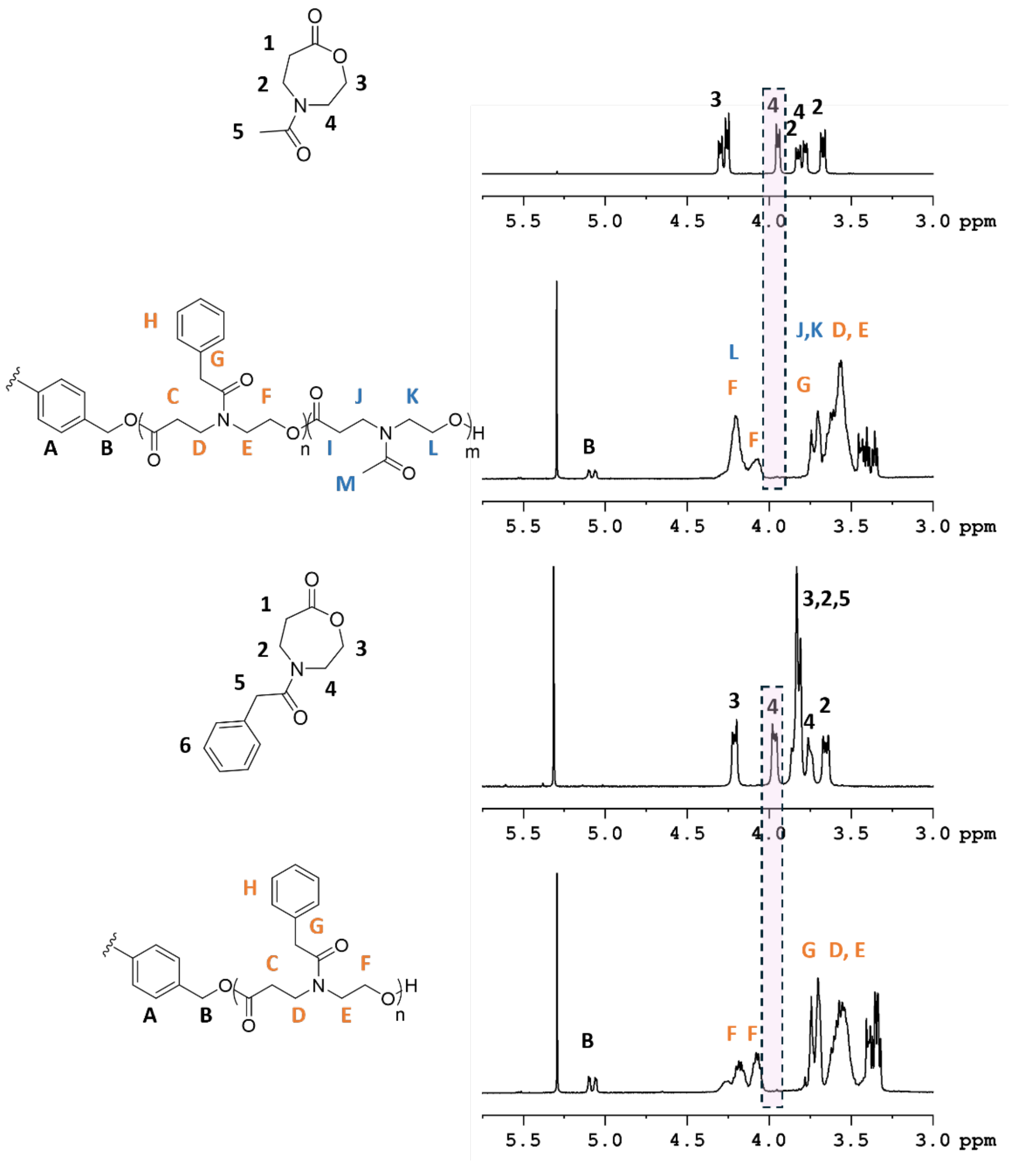

Figure S5: *Overlay of ^{1}H NMR spectra of reaction aliquots (Table 1, run 1) and monomers in* $CDCl_3$ *(enlargement between 3 and 5.75 ppm). From bottom to top: polymerization of* OxP_{Bn} *(first block);* OxP_{Bn}*; polymerization of* OxP_{Me} *(second block);* OxP_{Me}*.*

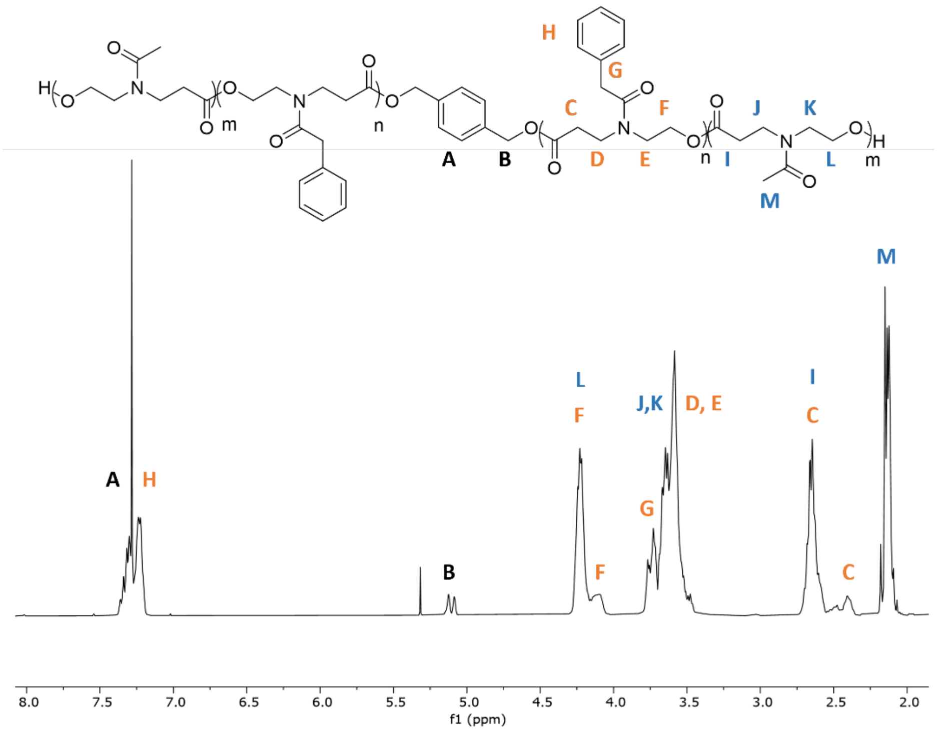

***Figure S6**: ^{1}H NMR of **P(OxP$_{Me}$)$_8$-b-P(OxP$_{Bn}$)$_8$-b-P(OxP$_{Me}$)$_8$** triblock copolymer in $CDCl_3$ (Table 1, run 2).*

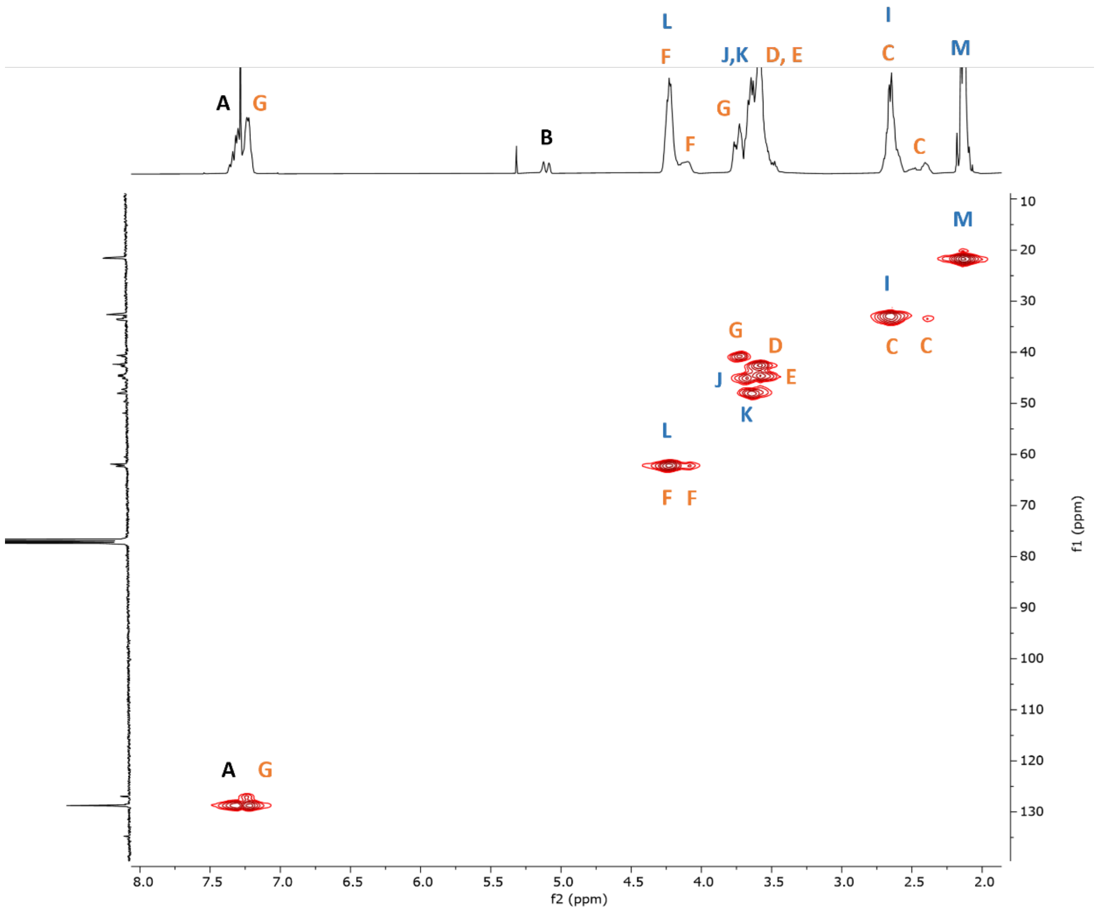

***Figure S7**: ^{1}H-^{13}C HSQC NMR of **P(OxP$_{Me}$)$_8$-b-P(OxP$_{Bn}$)$_8$-b-P(OxP$_{Me}$)$_8$** triblock copolymer in $CDCl_3$ (Table 1, run 2).*

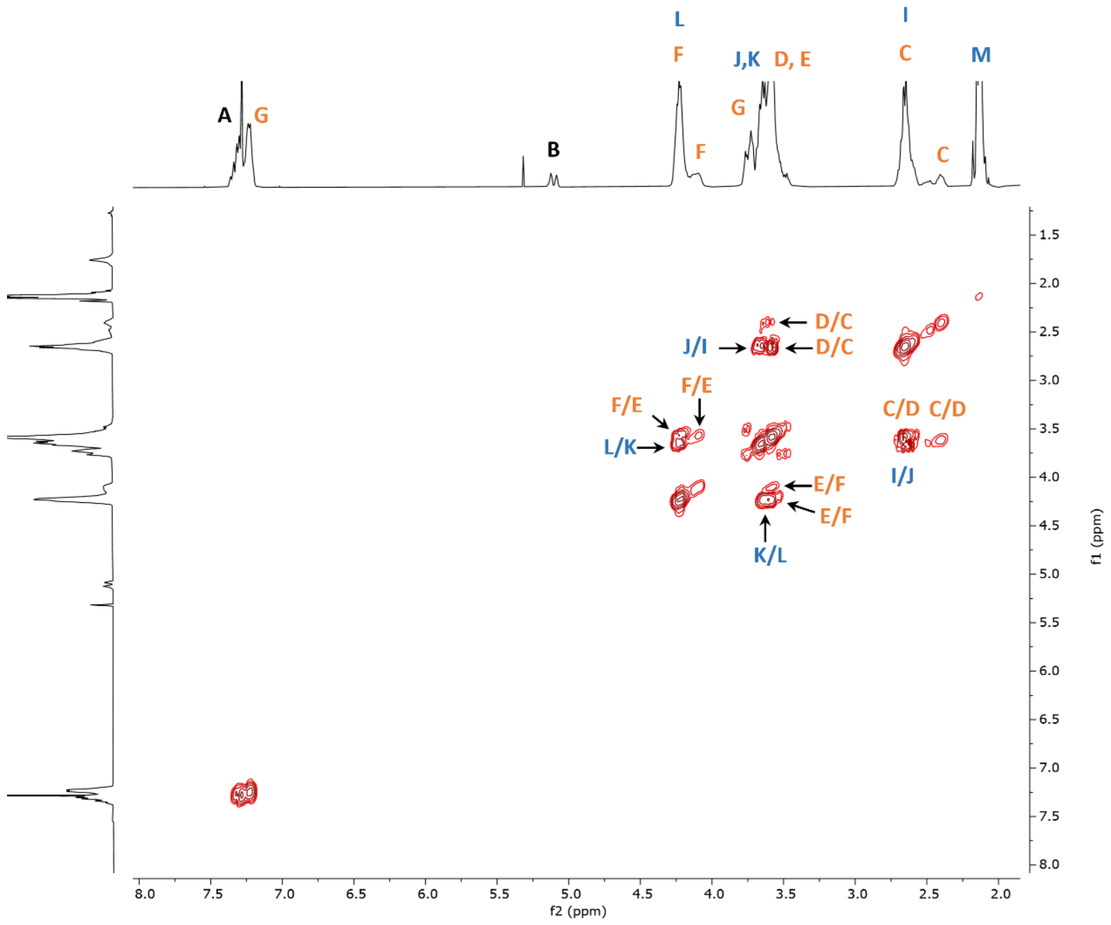

***Figure S8**: ^{1}H-^{1}H COSY NMR of P**(OxP$_{Me}$)$_8$-b-P(OxP$_{Bn}$)$_8$-b-P(OxP$_{Me}$)$_8$** triblock copolymer in $CDCl_3$ (Table 1, run 2).*

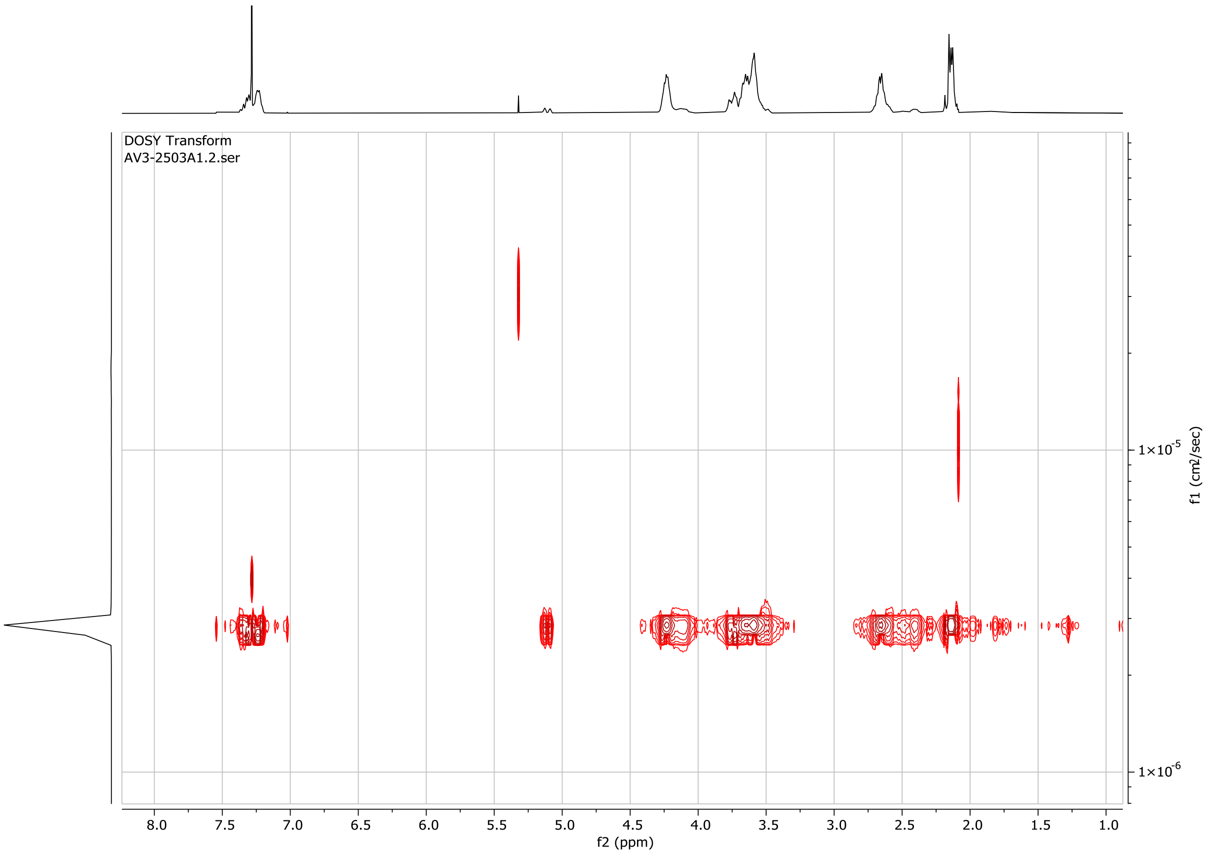

***Figure S9**: ^{1}H DOSY NMR of **$P(OxP_{Me})_8$-b-$P(OxP_{Bn})_8$-b-$P(OxP_{Me})_8$** triblock copolymer in $CDCl_3$ (Table 1, run 2).*

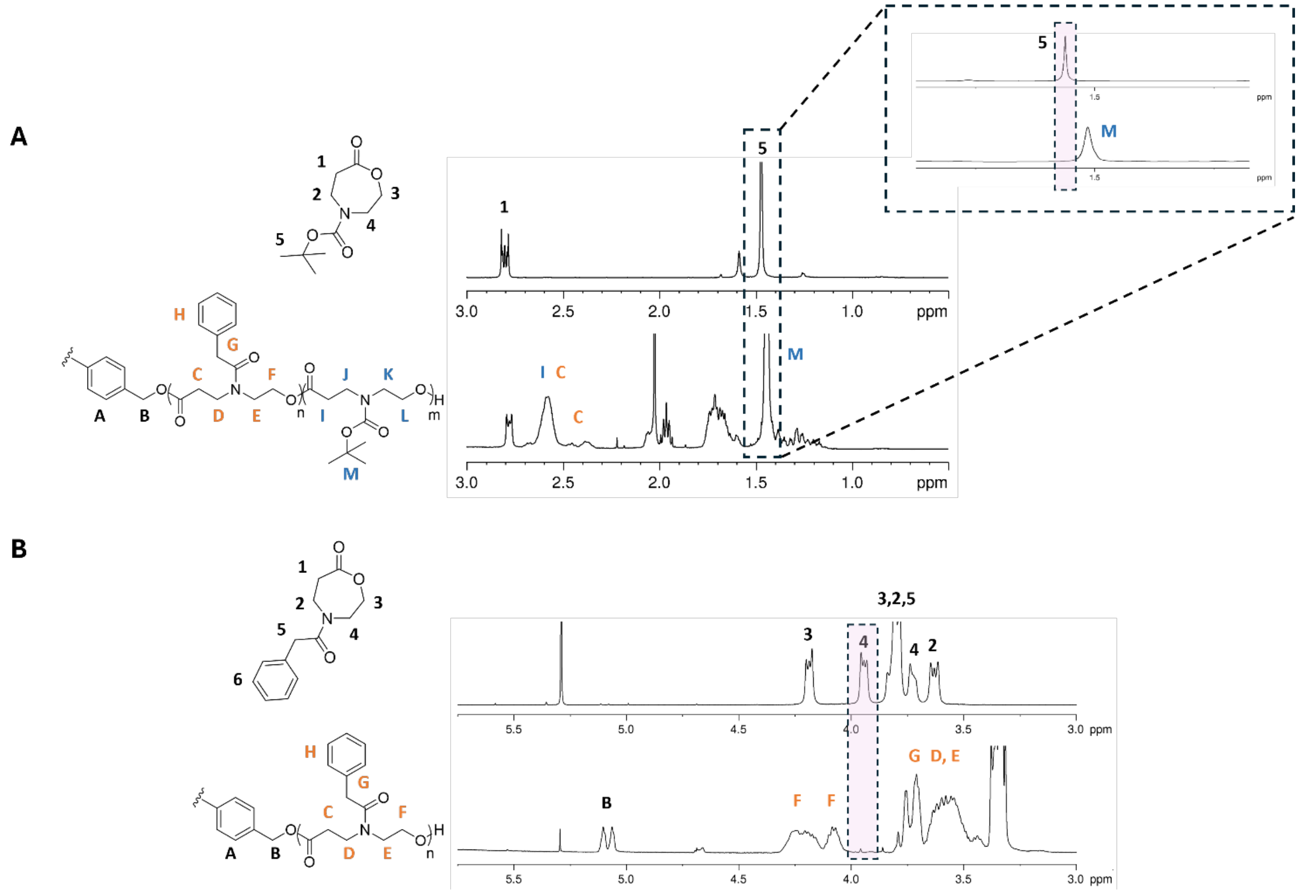

Figure S10: *Overlays of ^{1}H NMR spectra of reaction aliquots (Table 1, run 6 before deprotection) and monomers in $CDCl_3$. A) From bottom to top: polymerization of OxP_{Bn} (first block, enlargement between 3 and 5.75 ppm); OxP_{Bn}. B) From bottom to top: polymerization of OxP_{Boc} (second block, enlargement between 0.5 and 3 ppm); OxP_{Boc}.*

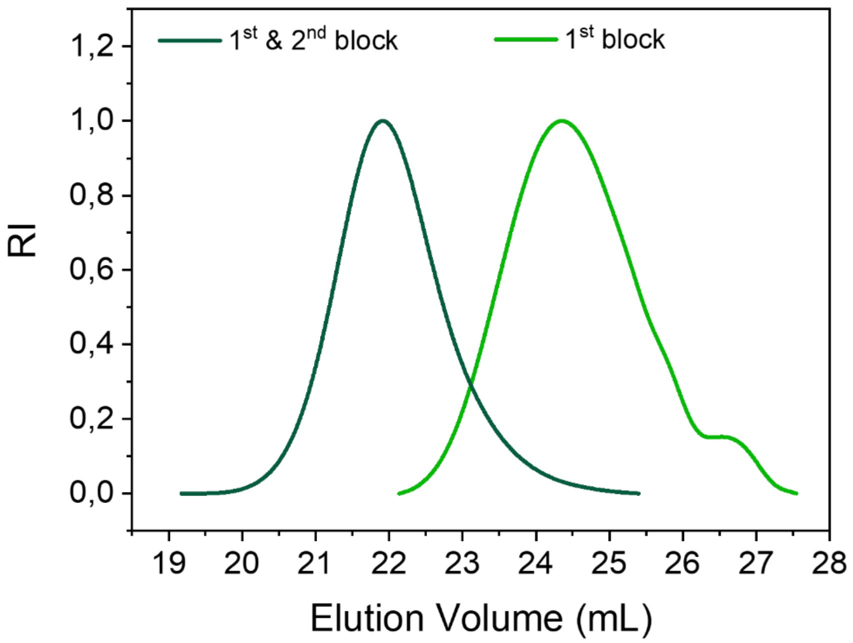

Figure S11: *SEC elution traces of* $\boldsymbol{P(OxP_{Boc})_8\text{-}b\text{-}P(OxP_{Bn})_8\text{-}b\text{-}P(OxP_{Boc})_8}$ *(Table 1, run 6 before deprotection) triblock copolymer synthesis (RI signal, DMF, standard: PMMA). First block (light green) and final copolymer (dark green).*

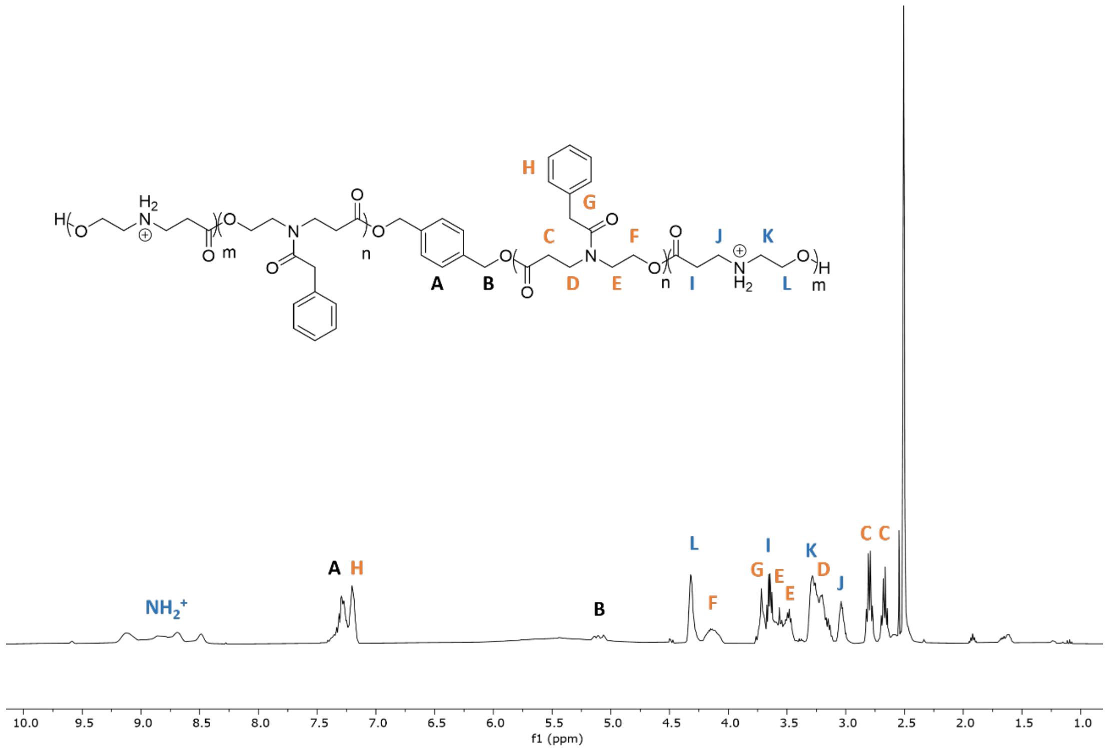

Figure S12: *^{1}H NMR of* $\boldsymbol{P(OxP_{NH2^+})_8\text{-}b\text{-}P(OxP_{Bn})_8\text{-}b\text{-}P(OxP_{NH2^+})_8}$ *triblock copolymer in DMSO-d_6 (Table 1, run 6).*

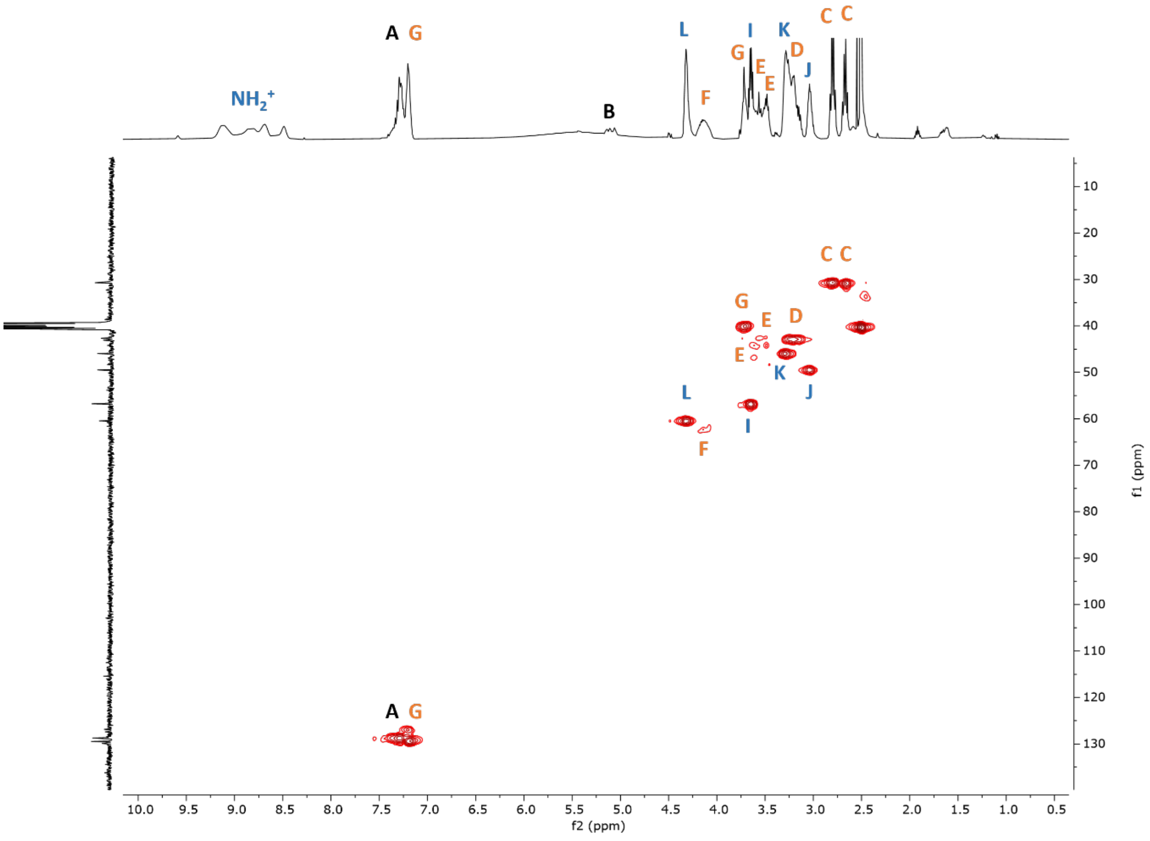

Figure S13: *^{1}H-^{13}C HSQC NMR of P(**OxP$_{NH2^+}$**)$_8$-**b**-P(**OxP$_{Bn}$**)$_8$-**b**-P(**OxP$_{NH2^+}$**)$_8$ triblock copolymer in DMSO-d$_6$ (Table 1, run 6).*

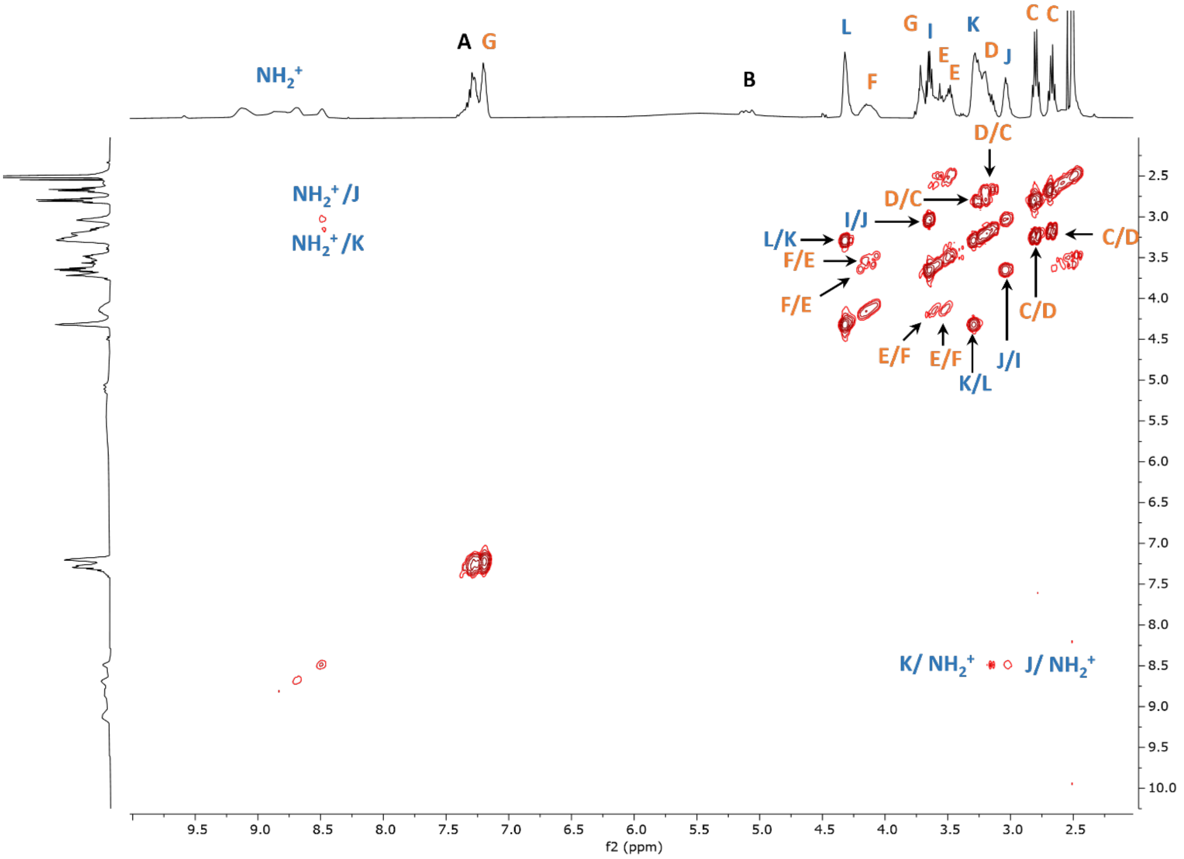

Figure S14: *^{1}H-^{1}H COSY NMR of* ***P(OxP$_{NH2^+}$)$_8$-b-P(OxP$_{Bn}$)$_8$-b-P(OxP$_{NH2^+}$)$_8$*** *triblock copolymer in DMSO-d$_6$ (Table 1, run 6).*

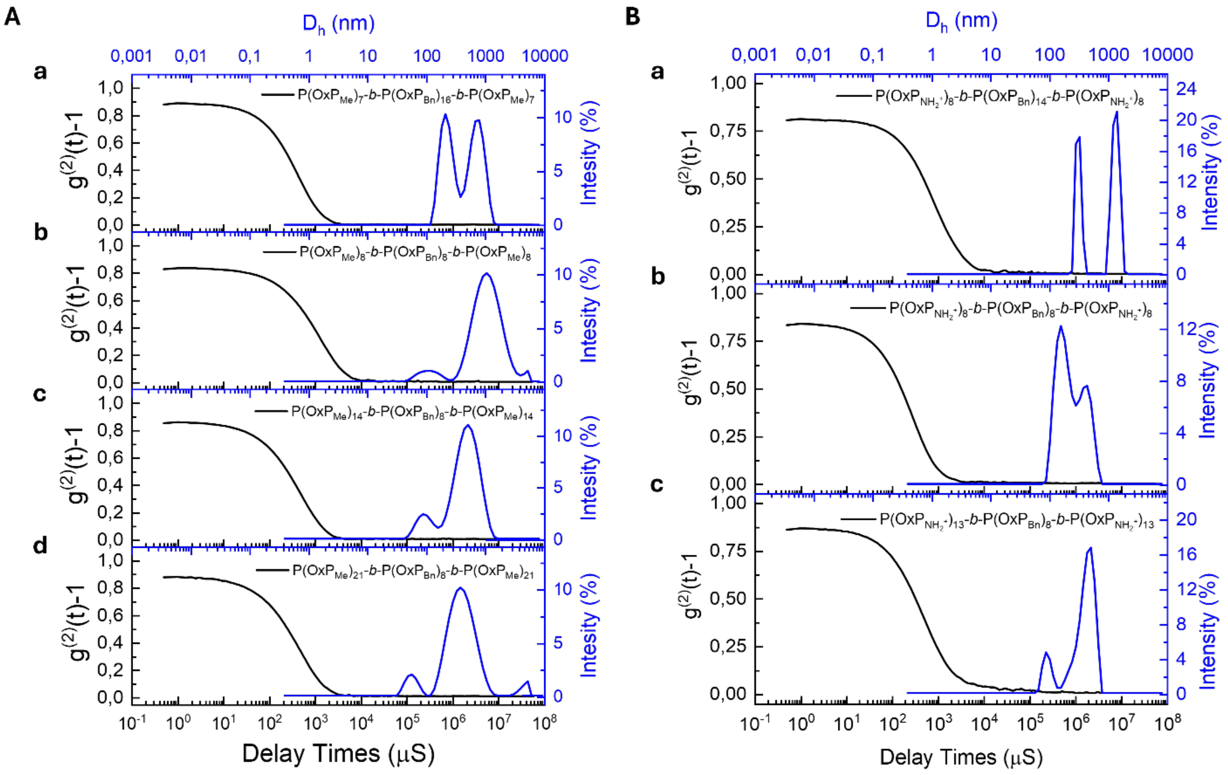

Figure S15: *DLS correlogram (black) and intensity weighted size distribution (blue) of A) P(OxP$_{Me}$)-b-P(OxP$_{Bn}$)-b-P(OxP$_{Me}$) and B) P(OxP$_{NH2+}$)-b-P(OxP$_{Bn}$)-b-P(OxP$_{NH2+}$) triblock copolymers. A)a.* ***P(OxP$_{Me}$)$_7$-b-P(OxP$_{Bn}$)$_{16}$-b-P(OxP$_{Me}$)$_7$*** *(Table 1, run 1). A)b.* ***P(OxP$_{Me}$)$_8$-b-P(OxP$_{Bn}$)$_8$-b-P(OxP$_{Me}$)$_8$*** *(Table 1, run 2). A)c.* ***P(OxP$_{Me}$)$_{14}$-b-P(OxP$_{Bn}$)$_8$-b-P(OxP$_{Me}$)$_{14}$*** *(Table 1, run 3). A)d.* ***P(OxP$_{Me}$)$_{21}$-b-P(OxP$_{Bn}$)$_8$-b-P(OxP$_{Me}$)$_{21}$*** *(Table 1, run 4). B)a.* ***P(OxP$_{NH2^+}$)$_8$-b-P(OxP$_{Bn}$)$_{14}$-b-P(OxP$_{NH2^+}$)$_8$*** *(Table 1, run 5). B)b.* ***P(OxP$_{NH2^+}$)$_8$-b-P(OxP$_{Bn}$)$_8$-b-P(OxP$_{NH2^+}$)$_8$*** *(Table 1, run 6). B)c.* ***P(OxP$_{NH2^+}$)$_{13}$-b-P(OxP$_{Bn}$)$_8$-b-P(OxP$_{NH2^+}$)$_{13}$*** *(Table 1, run 7).*

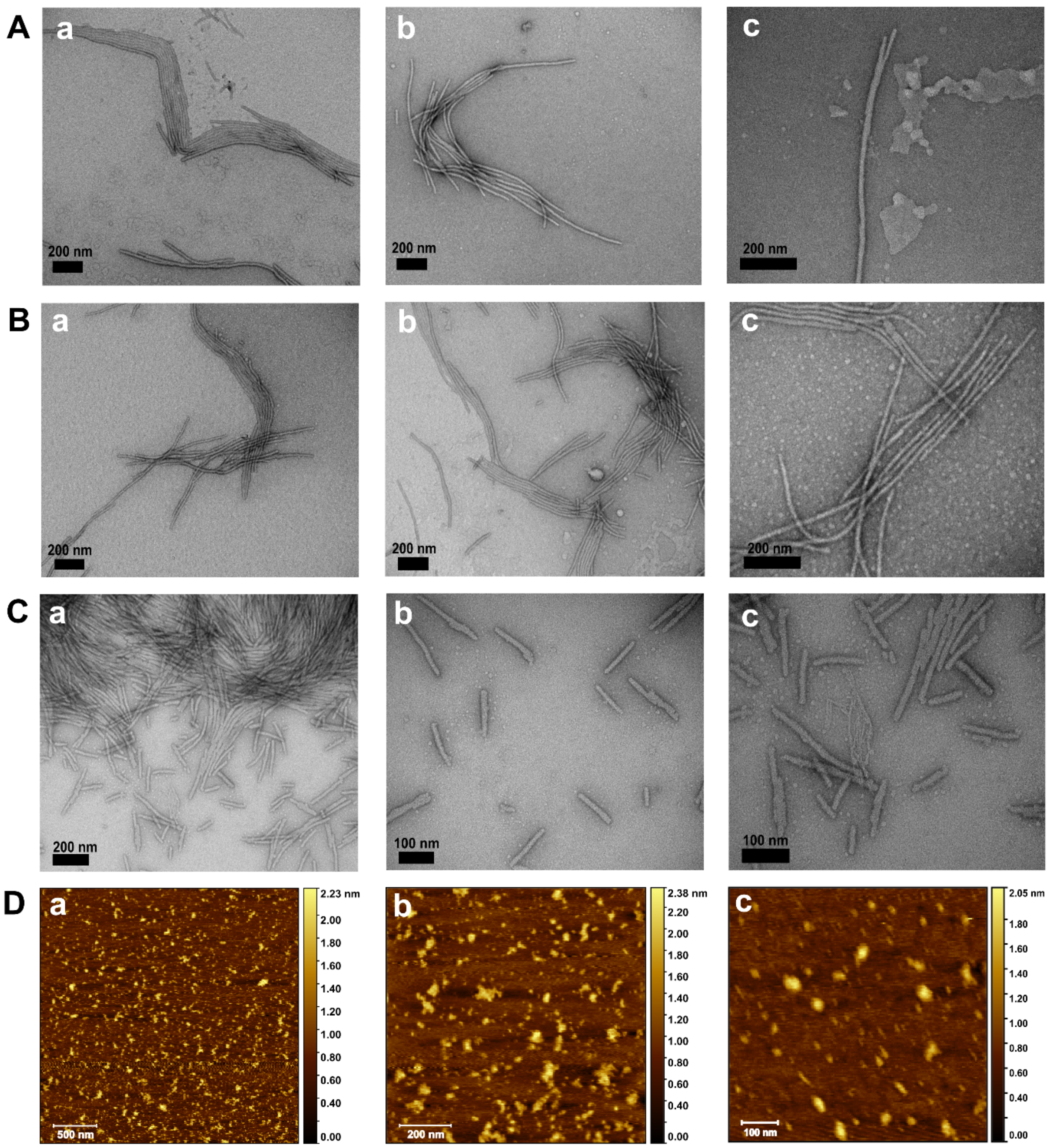

Figure S16: *TEM and AFM images of P(OxP$_{Me}$)-b-P(OxP$_{Bn}$)-b-P(OxP$_{Me}$) triblock copolymers. A)a-c. Selection of TEM images of* ***P(OxP$_{Me}$)$_{21}$-b-P(OxP$_{Bn}$)$_8$-b-P(OxP$_{Me}$)$_{21}$*** *(Table 1, run 4). Scale bars are: 200 nm, 200 nm, and 100 nm, respectively. B)a-c. Selection of TEM images of* ***P(OxP$_{Me}$)$_{14}$-b-P(OxP$_{Bn}$)$_8$-b-P(OxP$_{Me}$)$_{14}$*** *(Table 1, run 3). Scale bars are: 200 nm, 200 nm, and 100 nm, respectively. C)a-c. Selection of TEM images of* ***P(OxP$_{Me}$)$_8$-b-P(OxP$_{Bn}$)$_8$-b-P(OxP$_{Me}$)$_8$*** *(Table 1, run 2). Scale bars are: 200 nm, 100 nm, and 50 nm, respectively. D)a-c. Selection of AFM images of* ***P(OxP$_{Me}$)$_7$-b-P(OxP$_{Bn}$)$_{16}$-b-P(OxP$_{Me}$)$_7$*** *(Table 1, run 1). Scale bars are: 500 nm, 200 nm, and 100 nm, respectively.*

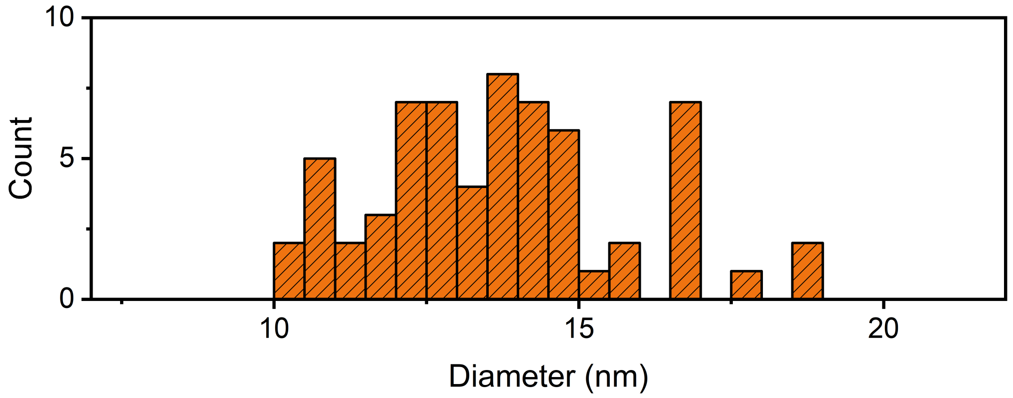

Figure S17**: Diameter of 14 ± 2 nm of* ***P(OxP$_{Me}$)$_8$-b-P(OxP$_{Bn}$)$_8$-b-P(OxP$_{Me}$)$_8$ *(Table 1, run 2) nanostructures determined from TEM images.*

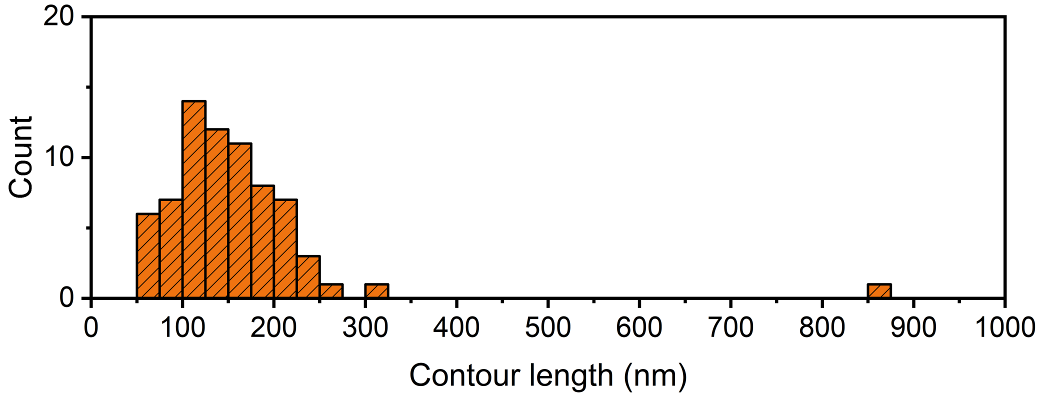

Figure S18**: Contour length of 156 ± 100 nm of* ***P(OxP$_{Me}$)$_8$-b-P(OxP$_{Bn}$)$_8$-b-P(OxP$_{Me}$)$_8$ *(Table 1, run 2) nanostructures determined from TEM images.*

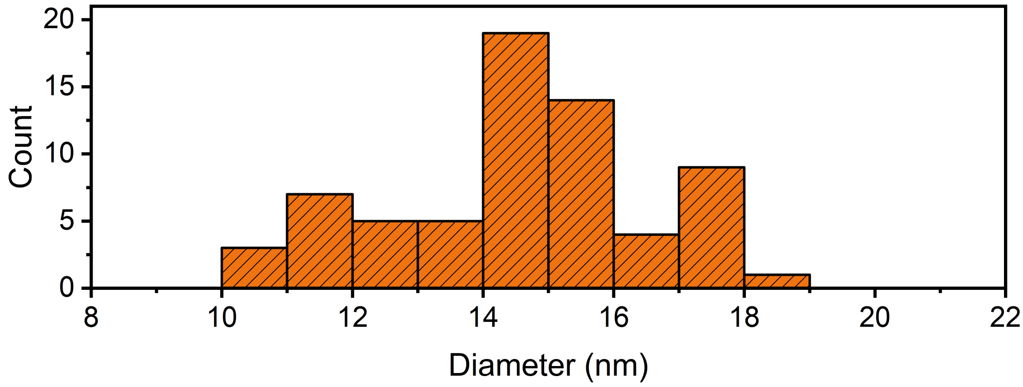

Figure S19**: Diameter of 15 ± 2 nm of* ***P(OxP$_{Me}$)$_{14}$-b-P(OxP$_{Bn}$)$_8$-b-P(OxP$_{Me}$)$_{14}$ *(Table 1, run 3) nanostructures determined from TEM images.*

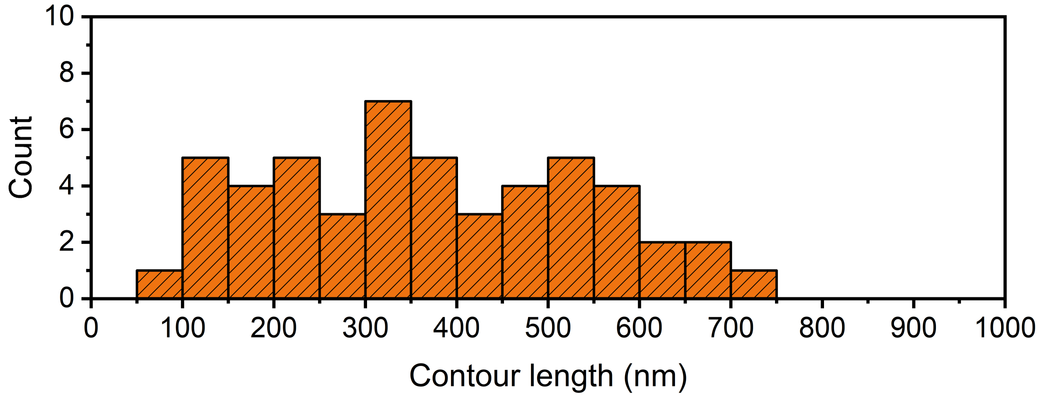

Figure S20: *Contour length of 372 ± 175 nm of* ***P(OxP$_{Me}$)$_{14}$-b-P(OxP$_{Bn}$)$_{8}$-b-P(OxP$_{Me}$)$_{14}$*** *(Table 1, run 3) nanostructures determined from TEM images.*

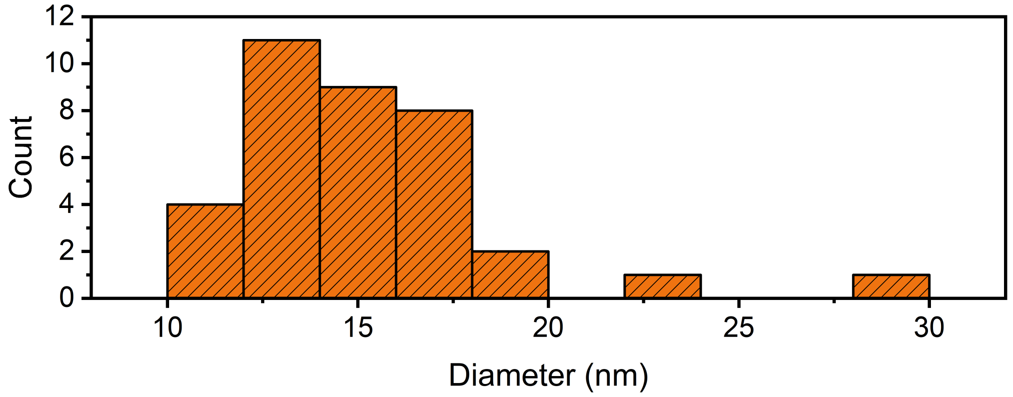

Figure S21: *Diameter of 15 ± 4 nm of* ***P(OxP$_{Me}$)$_{21}$-b-P(OxP$_{Bn}$)$_{8}$-b-P(OxP$_{Me}$)$_{21}$*** *(Table 1, run 4) nanostructures determined from TEM images.*

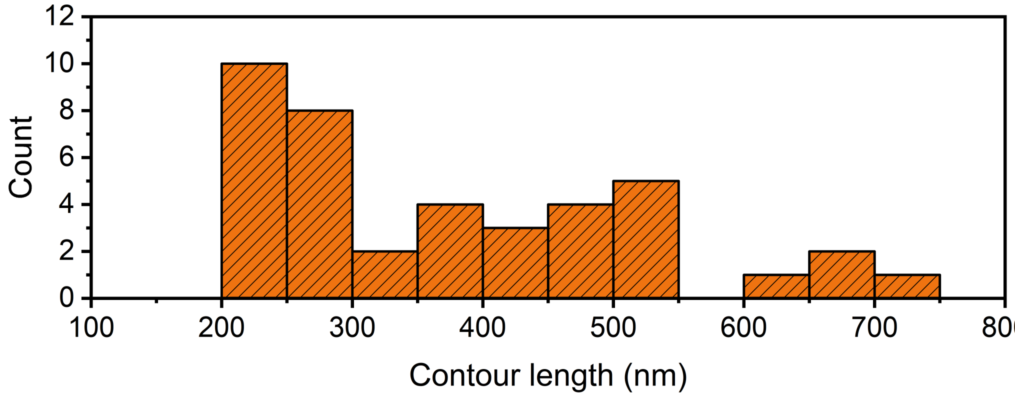

Figure S22: *Contour length of 378 ± 122 nm of* ***P(OxP$_{Me}$)$_{21}$-b-P(OxP$_{Bn}$)$_{8}$-b-P(OxP$_{Me}$)$_{21}$*** *(Table 1, run 4) nanostructures determined from TEM images.*

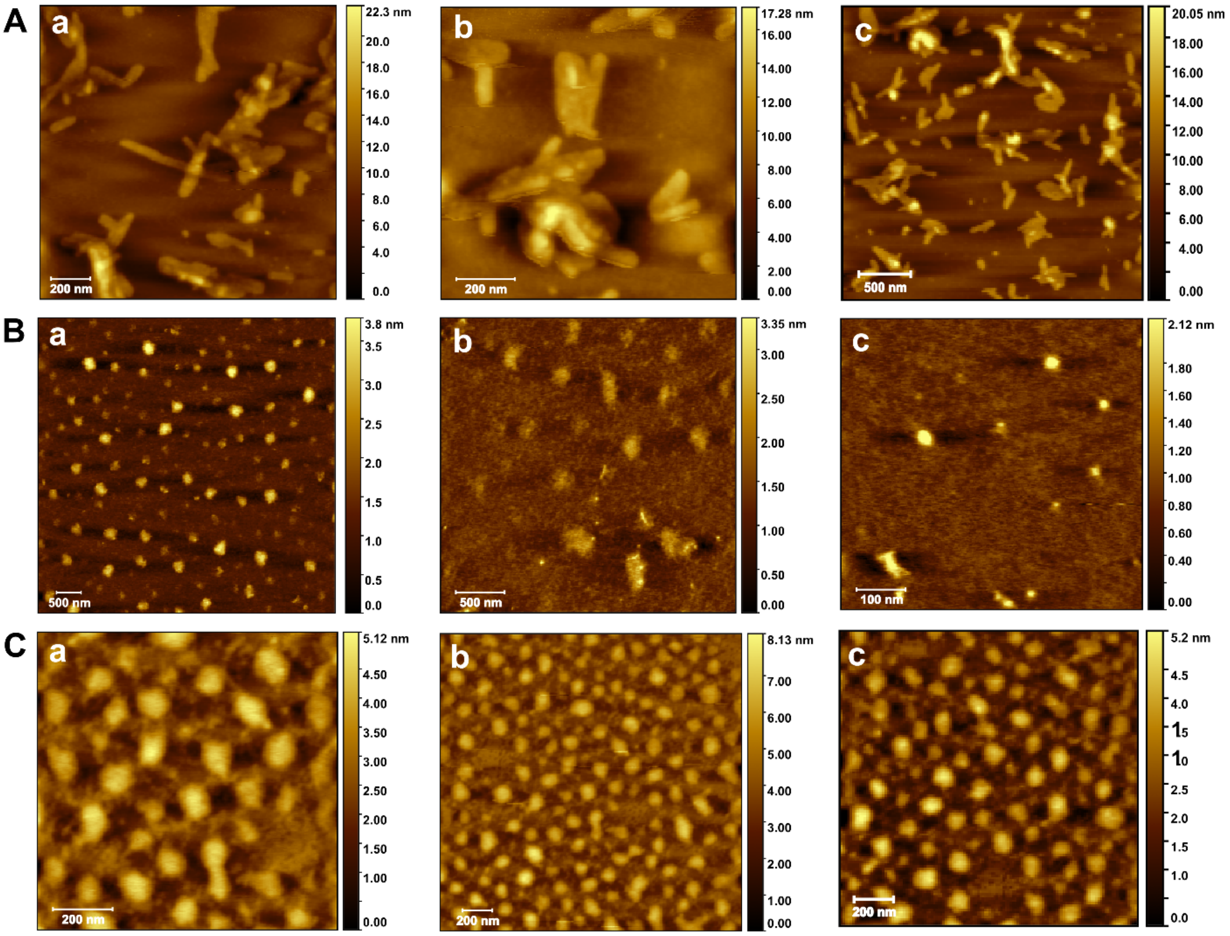

***Figure S23**: AFM images of $P(OxP_{NH2+})$-b-$P(OxP_{Bn})$-b-$P(OxP_{NH2+})$ triblock copolymers. A)a-c. Selection of images of **$P(OxP_{NH2^+})_{13}$-b-$P(OxP_{Bn})_{8}$-b-$P(OxP_{NH2^+})_{13}$** (Table 1, run 7). Scale bars are: 500 nm, 400 nm, and 200 nm, respectively. B)a-c. Selection of images of **$P(OxP_{NH2^+})_{8}$-b-$P(OxP_{Bn})_{8}$-b-$P(OxP_{NH2^+})_{8}$** (Table 1, run 6). Scale bars are: 500 nm, 500 nm, and 100 nm, respectively. C)a-c. Selection of images of **$P(OxP_{NH2^+})_{8}$-b-$P(OxP_{Bn})_{14}$-b-$P(OxP_{NH2^+})_{8}$** (Table 1, run 5). Scale bars are: 100 nm, 200 nm, and 500 nm, respectively.*

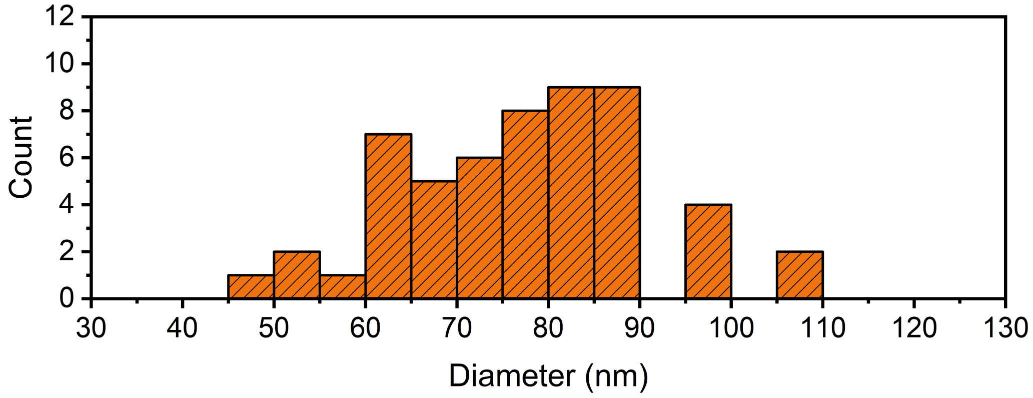

***Figure S24**: Diameter of 77 ± 13 nm of* **P(OxP$_{NH2^+}$)$_8$-b-P(OxP$_{Bn}$)$_{14}$-b-P(OxP$_{NH2^+}$)$_8$** *(Table 1, run 5) nanostructures determined from AFM images.*

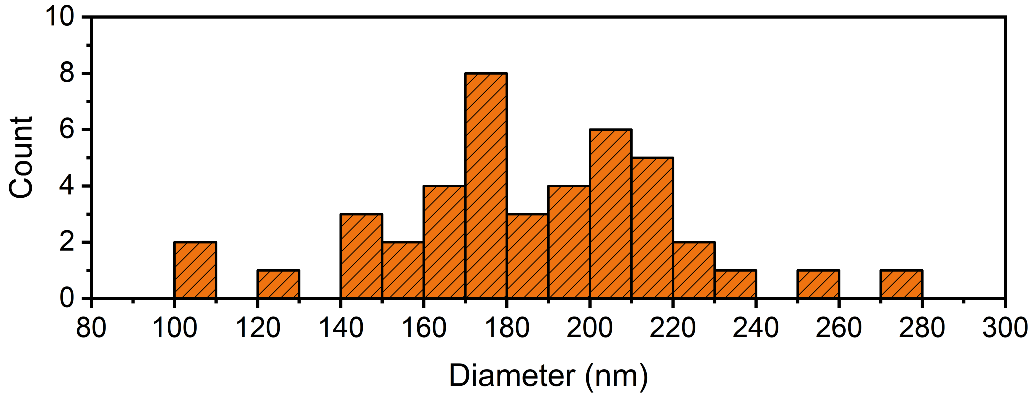

***Figure S25**: Diameter of 186 ± 34 nm of* **P(OxP$_{NH2^+}$)$_8$-b-P(OxP$_{Bn}$)$_8$-b-P(OxP$_{NH2^+}$)$_8$** *(Table 1, run 6) nanostructures determined from AFM images.*

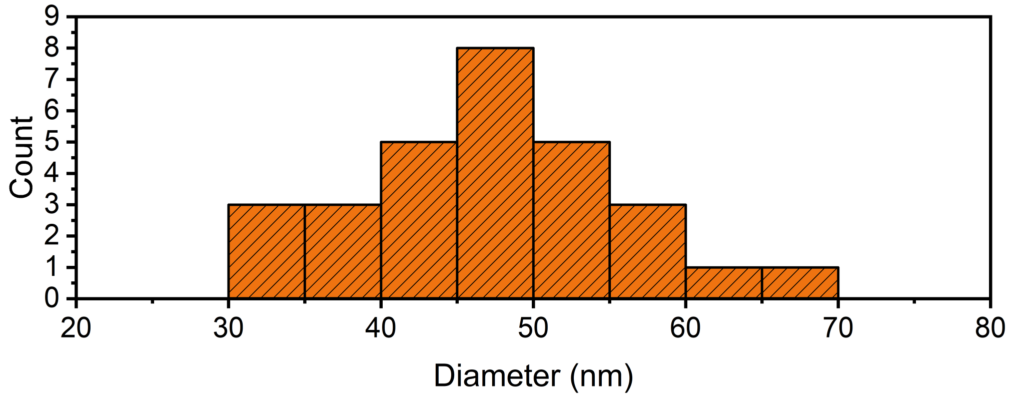

***Figure S26**: Diameter of 47 ± 9 nm of* **P(OxP$_{NH2^+}$)$_{13}$-b-P(OxP$_{Bn}$)$_8$-b-P(OxP$_{NH2^+}$)$_{13}$** *(Table 1, run 7) nanostructures determined from AFM images.*

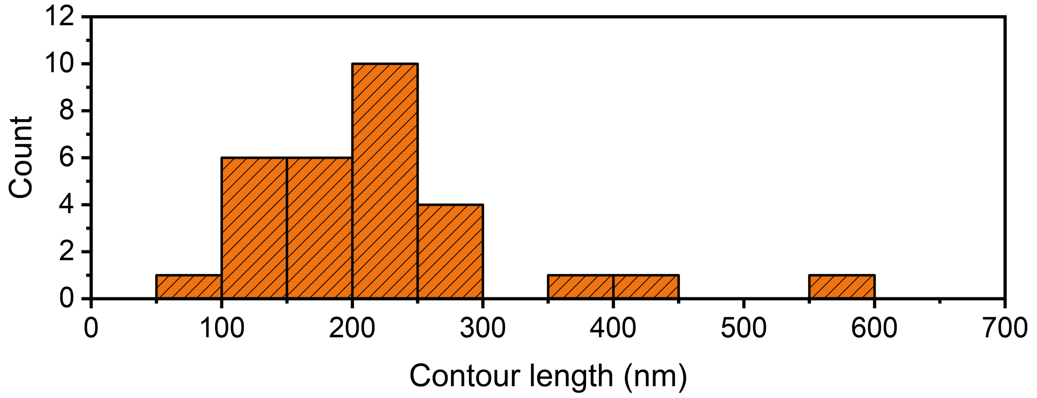

Figure S27: *Contour length of 220 ± 100 nm of* **P(OxP$_{NH2^+}$)$_{13}$-b-P(OxP$_{Bn}$)$_{8}$-b-P(OxP$_{NH2^+}$)$_{13}$** *(Table 1, run 7) nanostructures determined from AFM images.*

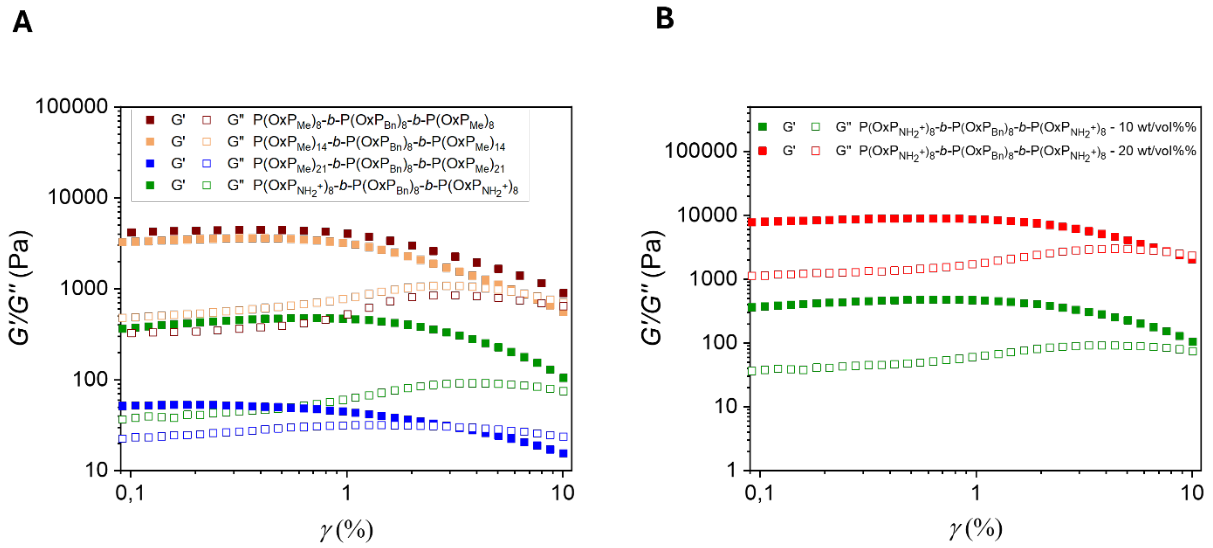

Figure S28: *Amplitude sweeps of triblock copolymers hydrogels. A) 10 wt% hydrogels using* **P(OxP$_{Me}$)$_8$-b-P(OxP$_{Bn}$)$_8$-b-P(OxP$_{Me}$)$_8$** *(Table 1, run 2),* **P(OxP$_{Me}$)$_{14}$-b-P(OxP$_{Bn}$)$_8$-b-P(OxP$_{Me}$)$_{14}$** *(Table 1, run 3),* **P(OxP$_{Me}$)$_{21}$-b-P(OxP$_{Bn}$)$_8$-b-P(OxP$_{Me}$)$_{21}$** *(Table 1, run 4), and* **P(OxP$_{NH2^+}$)$_8$-b-P(OxP$_{Bn}$)$_8$-b-P(OxP$_{NH2^+}$)$_8$** *(Table 1, run 6). B) Comparison between 10 wt% (green) and 20 wt% (red) hydrogels using* **P(OxP$_{NH2^+}$)$_8$-b-P(OxP$_{Bn}$)$_8$-b-P(OxP$_{NH2^+}$)$_8$** *(Table 1, run 6).*

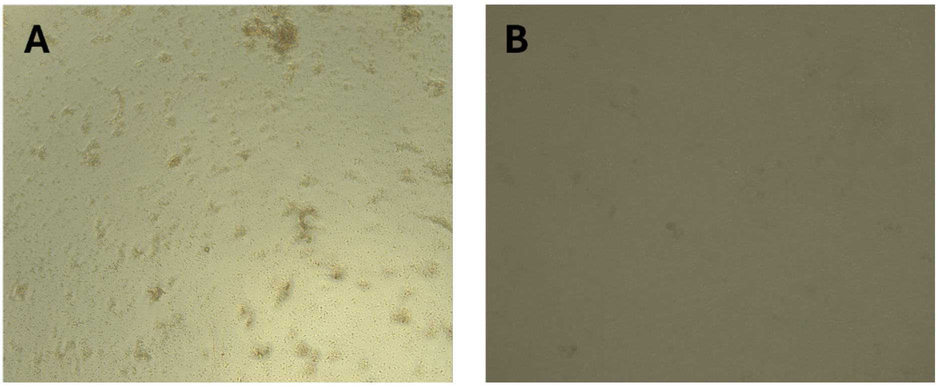

Figure S29: *Microscopy images of the spore proliferation control experiments. A) Experiment control 1. B) Experiment control 2.*
